# From Concrete to Code: A Survey of AI-Driven Transportation Infrastructure, Security, and Human Interaction

**DOI:** 10.3390/s26072219

**Published:** 2026-04-03

**Authors:** Nuri Alperen Kose, Kubra Kose, Fan Liang

**Affiliations:** Department of Computer Science, Sam Houston State University, Huntsville, TX 77341, USA; nxk022@shsu.edu (N.A.K.); kxg067@shsu.edu (K.K.)

**Keywords:** intelligent transportation systems, generative AI, roadside infrastructure, adversarial attacks, human–infrastructure interaction, cyber-physical security

## Abstract

The transition to AI-driven Cyber–Physical Systems has fundamentally reshaped transportation, introducing systemic risks that transcend traditional physical boundaries. Unlike prior reviews focused on isolated technological domains, this survey proposes a novel “End-to-End” analytical framework that models the causal propagation of vulnerabilities from physical sensing hardware to human cognitive responses. Synthesizing 140 research contributions (2017–2025), we evaluate the paradigm shift from deterministic control to Generative AI and Large Language Models (Transportation 5.0). To substantiate our framework, we introduce a structured cross-layer threat matrix and mathematically formalize the technology–cognition cascade, explicitly mapping how physical layer perturbations, such as optical jamming, bypass digital edge security to trigger hazardous behavioral reactions in human drivers. We conclude that ensuring the resilience of next-generation infrastructure requires a unified analytical architecture that formally bounds hardware constraints, algorithmic safety, and human trust.

## 1. Introduction

The transition from static infrastructure to smart city environments has fundamentally reshaped modern transportation systems. Contemporary Intelligent Transportation Systems (ITSs) are no longer limited to physical components such as roads and signage; instead, they operate as large-scale Cyber–Physical Systems (CPSs) that integrate Artificial Intelligence (AI), real-time sensing, and Vehicle-to-Everything (V2X) communication.

While these advances aim to reduce congestion, improve traffic efficiency, and enhance road safety, they simultaneously introduce new classes of systemic risk. The adoption of 5G and emerging 6G technologies enables ultra-low-latency communication and autonomous control, but it also expands the attack surface beyond physical access to include remote cyber intrusions, adversarial machine learning attacks, and large-scale false data injection. As roadside infrastructure increasingly operates with autonomous decision-making capabilities, such as dynamically adjusting signal timing or issuing real-time driver alerts, the reliability and integrity of the underlying data become directly linked to public safety and human life.

This survey presents a comprehensive analysis of the AI-driven transformation of roadside transportation infrastructure. In contrast to prior surveys that primarily focus on V2X communication protocols or isolated sensing modalities within autonomous vehicles, this work examines the infrastructure ecosystem as a cohesive and interdependent system. Specifically, the survey traces the end-to-end flow of information, beginning with physical sensing technologies such as radar, progressing through communication and aggregation components including Roadside Units (RSUs), advancing to control and decision-making mechanisms in traffic signal systems, and ultimately reaching the human driver through roadside alert and display systems.

The contributions of this survey can be summarized as follows:

- It synthesizes research across multiple infrastructure layers, including RSUs, radar sensing, traffic signal control, data integrity mechanisms, and roadside alert systems, enabling the identification of cross-layer vulnerabilities that are often overlooked in domain-specific studies.
- It examines the dual role of AI within smart transportation systems, highlighting its use as a defensive tool for intrusion detection, optimization, and resilience, while also analyzing its exploitation through adversarial examples, data poisoning, and model manipulation.
- It explicitly connects cyber and data-centric attacks on infrastructure components to their physical and cognitive consequences, with particular emphasis on driver perception, trust, and reaction behavior in response to manipulated information.
- It reviews emerging directions in intelligent infrastructure management, including the integration of Large Language Models (LLMs), generative intelligence, and digital twin technologies within next-generation transportation paradigms. This shift toward Transportation 5.0 represents a transition to Cyber–Physical–Social Systems (CPSSs), where generative AI and Parallel Intelligence harmonize hardware control with human behavioral patterns.

The remainder of this paper is organized as follows: Section 2 outlines the survey methodology, including the literature search strategy, gap analysis, and a comparison with existing reviews. Section 3 examines the evolution of V2X infrastructure and Edge Intelligence. Section 4 analyzes advances in Roadside Perception and multi-modal sensing. Section 5 details Intelligent Traffic Control, with a focus on generative AI and Large Language Models. Section 6 discusses Data Integrity, security threats, and adversarial defense mechanisms. Section 7 focuses on Human–Infrastructure Interaction and alert systems. Section 8 provides a cross-cutting analysis of layer-to-layer vulnerabilities, followed by future directions in Section 9. Finally, Section 10 concludes the survey by synthesizing the cross-layer implications of AI-driven infrastructure.

**Figure 1:**
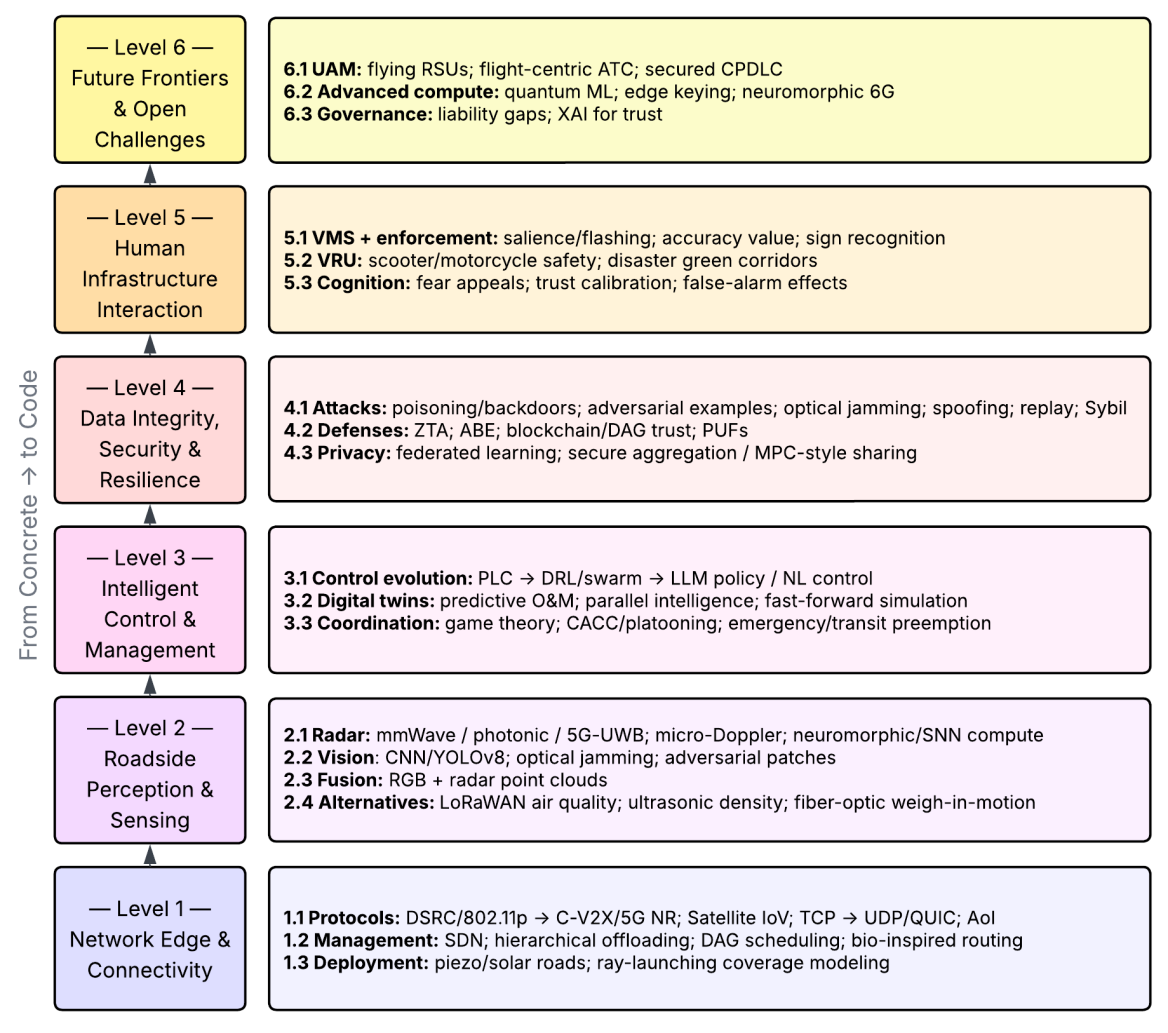
End-to-End analytical framework of intelligent transportation infrastructure. Rather than merely categorizing domains, this structure models the causal propagation of cross-layer vulnerabilities, demonstrating how physical-layer inputs constrain AI-driven control and ultimately dictate human cognitive responses.

Figure 1 illustrates the End-to-End scope of the paper by tracing the flow of information from physical infrastructure and connectivity (Levels 1–2), through intelligent control and security mechanisms (Levels 3–4), to human–infrastructure interaction and future systems (Levels 5–6). The layered structure emphasizes the transition from static Concrete assets to adaptive, software-defined Code-based intelligence, highlighting how sensing, computation, and decision-making progressively shift from hardware-bound components to data-driven and AI-enabled logic across the infrastructure stack.

## 2. Survey Methodology

In order to ensure a comprehensive review and minimize systematic sampling bias, we adopted a multistage search strategy focusing on the intersection of physical infrastructure, artificial intelligence (AI), and cybersecurity. We searched major databases including IEEE Xplore, ACM Digital Library, and ScienceDirect, supplemented by broad indexing platforms such as Google Scholar and targeted searches within specific relevant open-access journals (e.g., from the MDPI publisher). The search utilized keywords combining “Intelligent Transportation” and “Roadside Infrastructure” with “Generative AI,” “Large Language Models,” “Adversarial Attacks,” “Cyber–Physical Systems,” and “Digital Twins.” Furthermore, to bridge the gap between academic research and practical deployment, our selected studies explicitly incorporated essential industry reports, safety frameworks, and telecom standards, including 3rd Generation Partnership Project (3GPP) releases for 6G planning [1], International Organization for Standardization (ISO) safety standards (ISO 26262, ISO 21448) [2,3], IEEE standards [4], and National Highway Traffic Safety Administration (NHTSA) automated vehicle frameworks [5].

**Figure 2:**
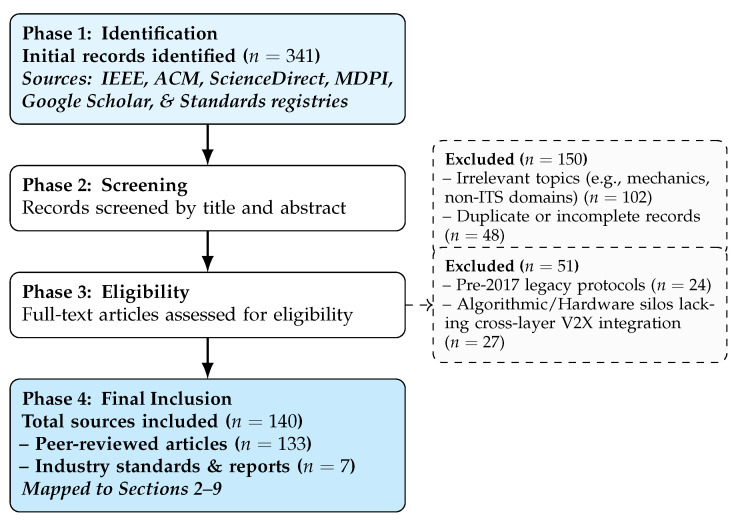
PRISMA flow diagram summarizing the literature selection process [6]. To ensure both scientific rigor and practical relevance, the final corpus of 140 sources is explicitly categorized into 133 peer-reviewed academic papers and 7 foundational industry standards/reports. Solid arrows and boxes indicate the progression of included records, while dashed arrows and gray boxes denote excluded records. Light blue and darker blue backgrounds highlight the initial identification and final inclusion phases, respectively. Bold text emphasizes phase titles and numerical outcomes, while italics indicate supplementary database sources and section mappings.

Figure 2 illustrates the multi-stage literature search methodology employed in this study. As shown in the diagram, the selection process filtered results from the listed sources to a final set of n=140 literature sources. To maintain scientific rigor while ensuring practical relevance, this final collection of studies is explicitly categorized into 133 peer-reviewed academic papers, which provide state-of-the-art algorithmic and architectural innovations, and 7 foundational industry standards and reports (e.g., ISO, IEEE, 3GPP, NHTSA, Federal Highway Administration (FHWA)), which define the regulatory and operational boundaries of real-world deployment. The search strategy for the peer-reviewed literature was specifically restricted to the period 2017–2025 to capture the emergence of disruptive technologies such as 6G, V2X advancements, and Large Language Models, identifying literature that addresses cross-layer dependencies.

To maintain the strict “End-to-End” scope of this survey, a specific exclusion criterion was applied during the full-text eligibility phase (Phase 3) for studies focusing on isolated algorithms. Specifically, 27 papers were excluded because they operated within algorithmic or hardware silos lacking a cross-layer V2X integration context. In our methodology, evaluating cross-layer vulnerabilities, such as how a physical sensor spoofing attack propagates to human cognitive responses, requires an underlying connected infrastructure. Studies that focused solely on algorithmic optimization for a single task (e.g., reinforcement learning for an isolated traffic light) or treated the physical sensor data as a static variable without addressing network communication were deemed outside the scope of this survey.

While numerous surveys address specific sub-domains of ITSs, few provide a complete, vertically integrated view spanning physical sensing hardware, communication and computation layers, and downstream human factors. Table 1 contrasts this survey with representative recent reviews and highlights the unique End-to-End scope of this work.

The unique End-to-End scope of this work compared to the existing literature is detailed in Table 1. This categorization highlights the breadth of the End-to-End scope, classifying the reviewed literature into five distinct infrastructure domains ranging from Network Edge architectures (Section 3) to Human Factors (Section 7).

**Figure 3:**
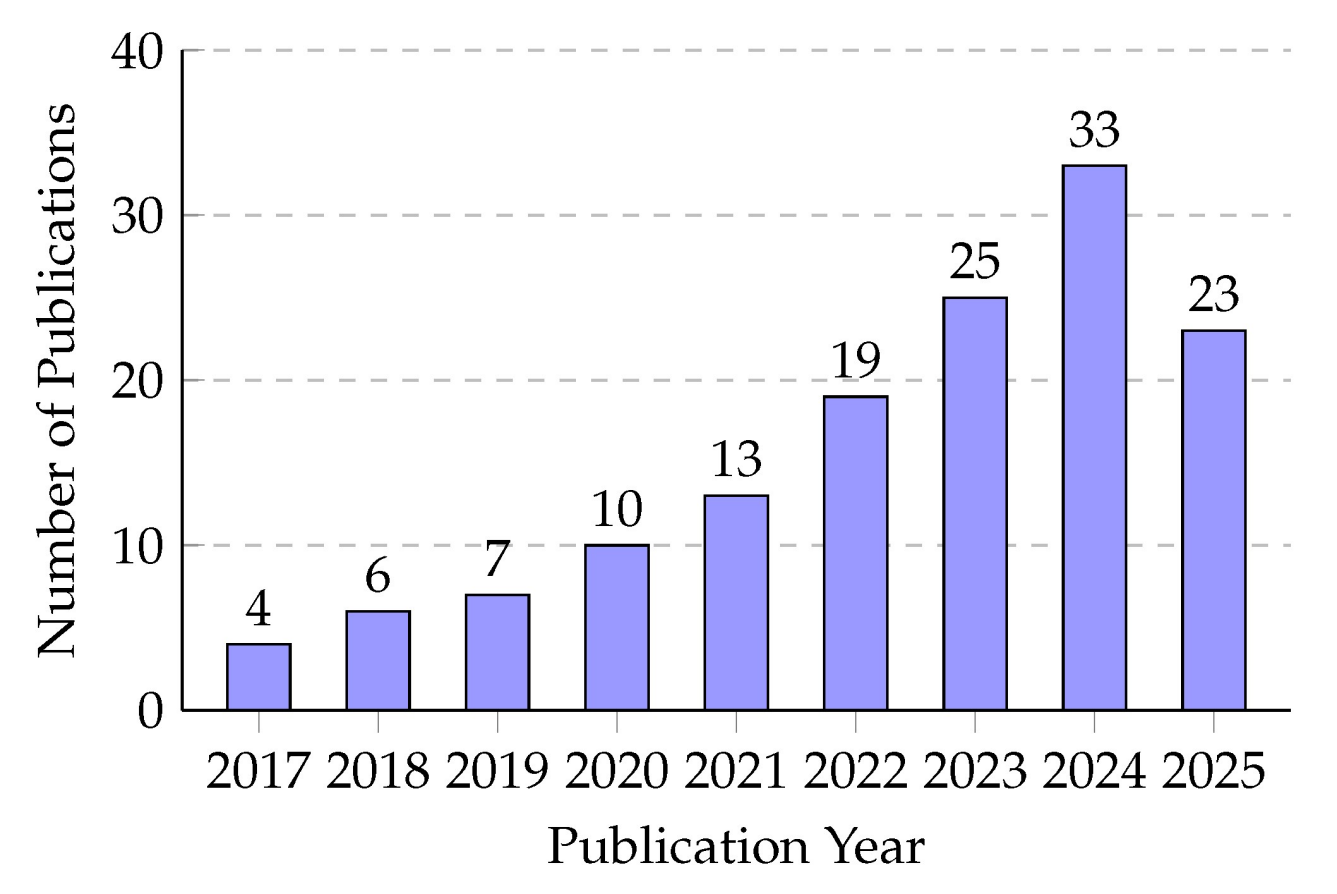
Temporal distribution of the 140 studies included in the survey (2017–2025). The results show a rapid increase in publications after 2022, reflecting growing research interest driven by advances in generative AI and V2X technologies.

To assess the developmental dynamics of the field, Figure 3 provides a statistical distribution of the included publications by year. The quantitative trend demonstrates a sharp acceleration in literature from 2022 onward, directly reflecting the integration of advanced 6G architectures and the paradigm shift toward Large Language Models in intelligent transportation.

Furthermore, Table 2 provides a quantitative classification of the methods and architectures synthesized across the 140 reviewed works. The numerical breakdown reveals a heavy focus on Traffic Control and AI logic (n=45, 32.1%), followed closely by Perception capabilities (n=36, 25.7%). Importantly, the statistical distribution demonstrates that Human Factors and cognitive interactions comprise a substantial 19.3% (n=27) of the literature, firmly establishing the human-infrastructure interface as a core pillar of this End-to-End survey. Additionally, the table reveals a heavy reliance on microscopic traffic simulators for control logic, highlighting a critical methodological gap in the use of high-fidelity co-simulation for security and perception validation.

### Gap Analysis and Uniqueness of This Survey

**Figure 4:**
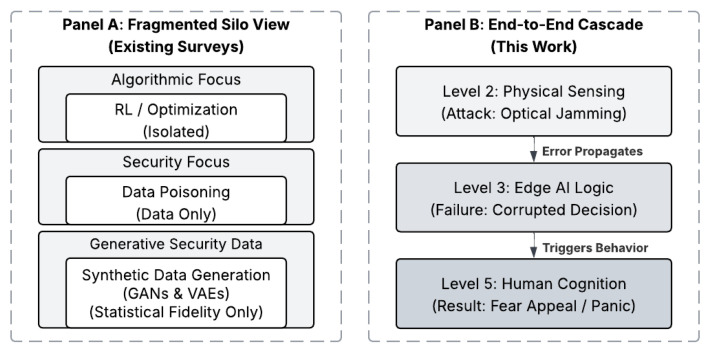
Visualizing the Research Gap: Fragmented Silos vs. End-to-End Integration. Panel A illustrates the “Silo Problem” in current literature, where critical domains are analyzed in isolation: Control Logic [9], Infrastructure Digital Twins [10], Data Security [8], Edge Computing [11], Physics-Informed Models [12] and Generative Security Data [21,22,23]. Panel B demonstrates the End-to-End Cascade approach of this survey, which bridges these layers to trace how physical sensing attacks propagate through algorithmic decision logic to trigger hazardous human cognitive responses.

Despite the rapid growth of literature on Intelligent Transportation Systems, existing surveys largely examine individual technological layers in isolation. As summarized in Table 1, prior reviews frequently concentrate either on algorithmic approaches for traffic flow optimization, such as swarm intelligence and reinforcement learning [9], or on narrowly scoped security threats, including data poisoning and related integrity attacks [8]. While digital twin-based frameworks have received increasing attention in recent survey work [10], the discussion is typically confined to infrastructure modeling and simulation. In particular, the literature does not explicitly connect (i) adversarial manipulation of physical sensing inputs that feed digital twins and control algorithms with (ii) the downstream behavioral and cognitive consequences of infrastructure outputs on human drivers. To visualize this critical distinction, Figure 4 contrasts the fragmented approach of existing literature with the unified methodology employed in this survey. As illustrated in Panel A, prior reviews typically operate within technological silos, optimizing control algorithms (e.g., Reinforcement Learning), analyzing data security (e.g., Poisoning) or focusing on synthetic data fidelity for intrusion detection in isolation, effectively treating the human operator as an external, static variable. Panel B demonstrates the End-to-End Cascade approach introduced in this work. This strict methodological boundary directly informed our literature screening process (Figure 2), mandating the exclusion of 27 papers that functioned perfectly within Panel A’s fragmented silos but lacked the cross-layer V2X connectivity required to support the cascade model in Panel B. By breaking these silos, we trace the causal propagation of threats vertically through the stack, showing how a physical layer attack (e.g., Optical Jamming) does not remain a hardware issue, but ascends to corrupt edge decision logic, ultimately triggering hazardous cognitive responses (e.g., Fear/Panic) in the driver.

This fragmented treatment creates a critical knowledge gap. Vulnerabilities originating at the physical sensing layer, such as radar spoofing or signal interference, can propagate through the communication and networking layer involving RSUs and V2X protocols, influence application-layer decision-making in traffic signal control or roadside alert generation, and ultimately manifest as unsafe reactions at the human interface, including panic braking or erosion of trust in system guidance. By synthesizing the complete sensing–computing–control–human pipeline, this survey provides an integrative perspective on how failures, attacks, and design limitations cascade across layers rather than remaining confined to a single subsystem.

The distinct contribution of this survey lies in its explicit focus on cross-layer interaction and End-to-End impact. Rather than terminating the analysis at algorithmic outputs, the survey traces how AI-driven decisions are delivered to users and how factors such as trust calibration, comprehension, and behavioral response determine system effectiveness. It further bridges protocol- and model-centric ITS research with physical sensing and platform-level constraints, including limitations in radar and vision systems as well as edge computing bottlenecks, enabling a unified assessment of threats and resilience across hardware and software boundaries. In addition, the survey incorporates emerging research directions from 2024 to 2025, including large language model-assisted policy and operations support and next-generation learning techniques for anomaly detection, situating future research challenges within a coherent layered framework.

## 3. Evolution of V2X Infrastructure and Architectures

The modernization of transportation infrastructure is predicated on the shift from isolated, hardware-centric components to connected, software-defined ecosystems. At the core of this transformation is the Roadside Unit (RSU), which has evolved from a simple data relay into an intelligent edge computing node capable of localizing decision-making. This section traces the architectural progression of vehicular networks, analyzing how the convergence of high-speed connectivity and edge intelligence is enabling the transition from static traffic management to dynamic, cooperative environments.

### 3.1. From DSRC to 5G and Cellular V2X (C-V2X)

Modern ITSs rely fundamentally on Vehicle-to-Everything (V2X) communication, in which RSUs function as critical edge components that connect highly dynamic vehicular networks with comparatively static core infrastructure. By aggregating sensor data, disseminating control information, and facilitating real-time coordination between vehicles and infrastructure, RSUs form the backbone of contemporary cooperative transportation architectures.

Early generations of V2X deployments were built upon Dedicated Short-Range Communications (DSRC/IEEE 802.11p), which primarily supported basic status message exchange such as Basic Safety Messages. While effective for short-range awareness, these technologies offer limited bandwidth and flexibility. As a result, the field has rapidly transitioned toward Cellular V2X (C-V2X) and 5G New Radio (NR) to accommodate high-throughput applications including cooperative perception and sensor sharing. This evolution, however, substantially enlarges the cyber attack surface. As emphasized by Nguyen et al., the increased connectivity inherent in these architectures introduces heightened exposure to impersonation, eavesdropping, and denial-of-service attacks within the Internet of Vehicles (IoV) ecosystem [24]. Looking forward, emerging architectures are extending V2X connectivity beyond terrestrial networks through the integration of satellite-based IoV, aiming to provide global coverage while introducing new authentication and trust challenges [25].

### 3.2. Operational Architectures: From Static Relays to Edge Intelligence

The operational role of RSUs is evolving rapidly as intelligent transportation systems transition from static communication relays to distributed edge intelligence platforms. Rather than serving solely as fixed forwarding devices, modern RSUs are increasingly designed as programmable, computation-aware infrastructure components. This evolution is driven by three fundamental challenges consistently identified in the literature: architectural rigidity, computational resource scarcity, and information freshness. Contemporary RSU architectures increasingly reflect a shift toward software-defined control, adaptive computation offloading, and transport mechanisms optimized for real-time situational awareness.

#### 3.2.1. Addressing Rigidity with Software-Defined Networking (SDN)

Traditional RSU deployments suffer from inherent hardware rigidity, making it difficult to upgrade protocols, reconfigure services, or adapt to highly dynamic traffic demands. To address these limitations, recent architectures increasingly decouple the control plane from the data plane using Software-Defined Networking (SDN) principles. This separation enables RSUs to operate as programmable edge clusters, where network behavior can be reconfigured through software rather than fixed-function hardware. By abstracting control logic, SDN-enabled RSUs support dynamic resource orchestration, allowing infrastructure operators to allocate 5G small cells to high-demand zones and rapidly adapt to traffic surges.

Nkenyereye et al. demonstrate that SDN-based RSU architectures improve support for high-bandwidth V2X services compared to legacy designs by enabling flexible traffic engineering and on-demand service provisioning [17]. However, while SDN improves architectural adaptability, it also introduces new vulnerabilities, most notably the emergence of centralized control points. This motivates the development of distributed and hierarchical control planes that preserve SDN’s flexibility while mitigating single points of failure and improving system resilience.

#### 3.2.2. Dynamic Resource Allocation and Offloading

As RSUs increasingly assume the role of edge computing nodes, static task offloading strategies become inadequate due to the unpredictable and dependency-driven nature of vehicular workloads. Unlike conventional linear execution pipelines, many vehicular applications consist of interdependent subtasks with variable latency constraints. Effectively managing these dependencies represents a primary architectural challenge for RSU-assisted computation.

To address this issue, Guan et al. propose a Directed Acyclic Graph (DAG)-based offloading strategy with dynamic parallel factor adjustment [18]. By explicitly modeling computational dependencies, this framework enables RSUs to adaptively decompose applications and optimize the degree of parallel execution across multiple edge clusters. In contrast to static or greedy offloading policies, DAG-based approaches improve resource utilization efficiency and reduce end-to-end latency, particularly for safety-critical services that demand predictable and low-latency responses. These results highlight the growing importance of dependency-aware scheduling mechanisms in next-generation RSU architectures.

#### 3.2.3. Optimizing Information Freshness Age of Information (AoI) and Transport Protocols

In safety-critical vehicular systems, the value of data is determined not only by delivery latency but also by its temporal relevance. Consequently, the AoI metric has emerged as a more appropriate performance indicator than throughput or delay alone. AoI captures the freshness of received updates and directly reflects the synchronization quality between physical traffic states and their digital representations.

Transmission protocols optimized for AoI prioritize newly generated beacons over queued packets, ensuring that the infrastructure’s operational world model remains aligned with real-world dynamics [26]. At the transport layer, this objective has accelerated the transition from Transmission Control Protocol (TCP)-based mechanisms to User Datagram Protocol (UDP)-based protocols such as QUIC, which are better suited for high-mobility and intermittently connected environments. Cluster-based analyses of QUIC traffic demonstrate that transport-layer parameters, including maximum transmission unit (MTU) size and segment configuration, influence flow dynamics and stability [27]. These findings indicate that adaptive transport-layer tuning is essential to prevent jitter, backlog accumulation, and stale-state propagation in volatile V2X networks, reinforcing the role of transport design as a first-class architectural concern in RSU-centric systems.

The synthesis of operational architectures highlights a fundamental latency–security trade-off. The convergence of SDN-driven control, dependency-aware offloading (DAG), and freshness-centric protocols (AoI) fundamentally transforms the RSU from a passive relay into a dynamic edge compute node. However, this transition introduces a critical flexibility–security trade-off. While decoupling the control plane via SDN and optimizing execution through DAGs maximizes resource utilization, it simultaneously concentrates network logic into software controllers, creating high-value targets for centralized Denial-of-Service attacks. Furthermore, the drive to minimize AoI through lightweight transport protocols like UDP and QUIC renders traditional batch-based security inspection infeasible due to latency constraints. This prioritization of freshness over inspection creates a “fast-path” vulnerability where malicious payloads may evade detection, a risk that necessitates the transition to the streaming-based Zero Trust architectures discussed in Section 6.3.

### 3.3. Intelligent Routing and Network Defense

While software-defined architectures establish global control and edge intelligence enhances computational capabilities, reliable data dissemination in vehicular environments ultimately depends on adaptive and resilient routing mechanisms. In sparse and highly mobile networks, traditional static routing tables and purely ad hoc forwarding strategies fail to maintain stable connectivity. Consequently, recent research increasingly emphasizes hybrid routing frameworks and intelligent control policies that integrate architectural coordination with learning-based and security-aware routing logic.

To address intermittent connectivity and fragmented topologies, Kumar and Raw propose a location-aware hybrid routing protocol (RC-LAHR) that adopts a hierarchical control structure [28]. Their approach offloads global route computation to a cloud layer while preserving low-latency forwarding decisions at the RSU. This division of responsibility enables infrastructure-assisted path optimization without sacrificing responsiveness at the network edge. Experimental evaluations demonstrate that this hierarchical design improves packet delivery ratios in disconnected and delay-tolerant scenarios compared to purely ad hoc routing schemes, highlighting the importance of multi-tier routing intelligence in RSU-supported vehicular systems.

In environments characterized by extreme topology volatility, bio-inspired optimization techniques have emerged as effective alternatives to deterministic routing strategies. Bijalwan et al. introduce an enhanced Ant Colony Optimization (ACO) framework combined with Fittest Node Clustering to stabilize route discovery in Vehicular Ad Hoc Networks [29]. By clustering nodes according to stability and energy metrics prior to pheromone-based path selection, the proposed method reduces the routing overhead associated with blind broadcasting while improving convergence speed. These results indicate that swarm-based heuristics offer practical mechanisms for maintaining routing efficiency under continuous network reconfiguration.

However, the openness and adaptivity of vehicular routing protocols also expose them to severe security threats. As a result, recent studies increasingly embed artificial intelligence directly into routing and forwarding logic to enable real-time attack detection and mitigation. ul Hassan et al. demonstrate that Artificial Neural Networks (ANNs) can analyze historical node behavior to identify Blackhole attacks, in which malicious relays silently discard packets [30]. Their learning-based framework enables RSUs to reroute traffic around compromised nodes with higher detection accuracy than conventional trust-based models. Similarly, to counter Wormhole attacks that distort routing metrics through adversarial tunneling, Kumar and Gupta introduce an RSU-assisted validation scheme that correlates hop counts with physical distance constraints [31]. By enforcing consistency between logical topology and physical reality, their approach effectively exposes malicious shortcut links and preserves routing integrity.

Together, these studies illustrate a broader transition from static forwarding mechanisms toward intelligent, security-aware routing ecosystems. In such architectures, RSUs not only coordinate network resources but also participate directly in learning-driven routing optimization and real-time threat response, reinforcing their emerging role as cognitive control points within smart transportation infrastructures. The synthesis of routing intelligence highlights a fundamental efficiency–integrity tension. The evolution of routing protocols, from static forwarding to hybrid and bio-inspired optimization, reveals a growing dependency on algorithmic trust. While mechanisms such as Ant Colony Optimization and hierarchical routing successfully maintain connectivity in volatile environments, their adaptive nature makes them inherently susceptible to topology poisoning. An adversary manipulating routing metrics can easily disrupt bio-inspired convergence or exploit hybrid handovers to execute wormhole or blackhole attacks, as demonstrated by the need for ANN-based detection logic. Consequently, modern routing layers can no longer function solely as transport mechanisms; they must integrate real-time verification logic. This creates a computational tension at the edge: the RSU must balance the processing overhead of AI-driven intrusion detection against the imperative of low-latency forwarding, limiting the complexity of security models that can be deployed on current hardware.

### 3.4. Physical Deployment Strategies

As Radio Frequency (RF) spectrum congestion intensifies in urban environments, RSU architectures must incorporate alternative physical layers. Hybrid routing protocols that integrate Visible Light Communication (VLC) with conventional RF have emerged as a robust solution. In this model, RSUs manage communication “zones” that alternate between directional RF and line-of-sight optical channels, allowing data traffic to be offloaded to the optical spectrum during RF saturation to ensure the delivery of emergency messages [32]. Furthermore, the functional scope of RSUs is extending beyond communication to active sensing. In remote or infrastructure-sparse regions, low-orbit satellites are utilized as orbiting RSUs, employing modular error-correction codes to secure long-distance links. On the ground, RSUs are being repurposed as radar sensors. Sensing-assisted frameworks allow the RSU to track vehicle distance and angle to estimate channel correlation, dynamically removing redundant pilot signals to improve downlink throughput [33].

#### 3.4.1. Sustainable and Energy-Harvesting Infrastructure

A critical barrier to widespread RSU deployment is the reliance on grid power. To address this, the “Coexistence” concept integrates ITSs with energy-harvesting road infrastructure. Piezoelectric and solar pavement technologies enable roads to harvest energy from solar radiation and mechanical traffic loads, effectively turning the road surface into a power plant for the RSU network. In this way, it reduces the operational expenditure of smart corridors while ensuring continuous power availability for edge sensing [34]. From a security perspective, energy autonomy creates resilience against grid-targeted cyber-physical attacks; however, it introduces a new dependency where physical damage to the pavement constitutes a Denial-of-Service (DoS) attack on the communication network.

#### 3.4.2. Propagation Modeling and Coverage Optimization (Ray Launching)

The effectiveness of high-frequency RSUs (mmWave/5G) is heavily dependent on line of sight. Deterministic propagation approaches using Ray Launching algorithms simulate complex multipath environments (e.g., parking lots) to optimize RSU placement. By modeling the interaction of 28 GHz signals with obstacles like parked vehicles and vegetation, these tools allow network planners to guarantee coverage “blind spots” are minimized before physical deployment [35]. Beyond logic, optimization extends to physical deployment. Strategic placement of RSUs, when combined with Variable Speed Limit dissemination, can actively smooth traffic flow and reduce vehicular emissions, adding an environmental sustainability metric to RSU network planning [36].

## 4. Roadside Perception and Multi-Modal Sensing

Reliable control logic depends fundamentally on accurate, real-time perception of the physical environment. Unlike autonomous vehicles, which rely on onboard sensors with limited fields of view, roadside infrastructure offers a broader, unbiased perspective of complex traffic scenarios. This section surveys the state of the art in roadside sensing, examining the trade-offs between the range and robustness of radar architectures versus the semantic richness of visual perception systems, and how these modalities are fused to overcome their respective physical limitations. Table 3, which provides a comparative analysis of roadside perception modalities, summarizes the perception technologies, including their specific security risks. It maps the operational advantages of technologies like mmWave radar and vision systems against their specific physical and cyber vulnerabilities, such as ghost targets and optical adversarial patches.

### 4.1. Advanced Radar Technologies

Among roadside sensing modalities, radar remains the standard for non-intrusive traffic monitoring due to its resilience against adverse weather and variable lighting conditions. However, the demand for higher resolution and object classification has necessitated a departure from traditional Doppler units toward high-bandwidth imaging technologies. The following subsections detail emerging radar architectures that bridge the gap between simple speed detection and high-fidelity spatial imaging.

#### 4.1.1. mmWave, Photonic, and UWB Radar Architectures

Millimeter-wave (mmWave) radar has become the dominant technology for non-intrusive traffic monitoring due to its robustness under adverse weather and lighting conditions. Recent research focuses on pushing the physical limits of radar resolution while improving processing efficiency to support real-time roadside deployment.

Conventional electronic radar systems are often constrained by bandwidth limitations that restrict achievable range resolution. To address these constraints, photonic radar architectures have emerged as a promising alternative. Photonic-based generation of Frequency-Modulated Continuous Wave signals with a bandwidth of 4 GHz achieves a range resolution of 7 cm. When combined with Support Vector Machine classifiers, this architecture enables reliable multi-target detection under complex traffic and weather conditions, outperforming traditional electronic radar solutions [38].

In parallel, radar sensing is increasingly integrated into Internet of Things edge architectures. 5G-connected Ultra-Wideband (UWB) radar platforms process data locally and transmit metrics via Message Queuing Telemetry Transport (MQTT) over 5G links, enabling real-time traffic monitoring without reliance on extensive wired infrastructure [43].

#### 4.1.2. Signal Processing: Neuromorphic Computing and Micro-Doppler Signatures

As radar resolution and sensing density increase, computational efficiency becomes a critical concern for edge deployment. Neuromorphic computing using Spiking Neural Networks (SNNs) processes asynchronous event-driven spikes rather than frame-based inputs. This biologically inspired approach offers significant energy efficiency advantages for radar signal processing at the edge, although practical challenges remain in converting continuous radar returns into discrete spike representations [44]. For traditional electronic radar systems, tracking high-speed targets introduces range walk effects. Applying the Keystone Transform effectively compensates for motion-induced energy smearing, improving target localization accuracy in 77 GHz traffic monitoring systems [45]. Beyond basic detection, accurately identifying the type of road user is essential for safety-critical traffic applications. Radar-based classification enables infrastructure to distinguish between pedestrians, cyclists, and vehicles, supporting differentiated responses within intelligent transportation systems.

Distinct traffic participants exhibit characteristic micro-motion patterns that manifest as unique micro-Doppler signatures. Transforming these time–frequency representations into spectrogram images allows Convolutional Neural Networks (CNNs) to classify objects with high accuracy, effectively differentiating vulnerable road users from motorized vehicles under diverse traffic conditions [46]. To further enhance spatial accuracy, replacing traditional geometric estimation with Random Forest regression models allows the system to learn non-linear relationships between measured radar features and true vehicle positions, resulting in improved localization performance [47].

### 4.2. AI-Driven Object Classification (YOLO, CNNs)

While radar provides range, vision provides semantics. Autonomous traffic-monitoring systems utilizing YOLOv8 architectures achieve high-precision pedestrian crossing detection by integrating motion sensors with deep learning vision. This allows for the real-time identification of “jaywalking” behaviors or stalled pedestrians, triggering immediate alerts to approaching connected vehicles [16]. The security implication of such high-speed detection is critical: if an adversary can execute a “pattern patch” attack to render a pedestrian invisible to YOLOv8 (Adversarial Example), the system fails to trigger the braking response, converting a digital evasion into a physical fatality. Traditional induction loops are being replaced by vision-based actuators. Traffic management systems utilizing YOLO (You Only Look Once) algorithms allow controllers to measure queue density and vehicle type in real time. By feeding this visual data into embedded controllers, signal phases are adjusted dynamically based on actual occupancy rather than fixed timers, maximizing throughput for heavy vehicles [39]. From a security perspective, relying on visual actuation expands the attack surface; if an attacker projects a video of an empty road into the camera sensor (Sensor Spoofing), the controller could dangerously shorten green times for actual traffic.

### 4.3. Multi-Modal Sensor Fusion

To capitalize on the distinct advantages of both modalities, infrastructure designers are increasingly integrating the robust kinematics of radar with the semantic richness of vision. This multi-modal fusion enhances overall system reliability and situational awareness, overcoming the physical limitations inherent to any single sensor.

#### 4.3.1. Integrating Radar, Camera, and Lidar Data

Deep fusion architectures combine roadside RGB camera data with sparse three-dimensional radar point clouds using feature pyramid networks. The Infra-3DRC-FusionNet framework demonstrates improved traffic participant detection by jointly exploiting complementary spatial and semantic information from both modalities [48]. Complementary studies highlight the benefits of deep learning-based fusion for pedestrian detection, showing that integrating depth and range cues with visual data reduces false positives in complex urban environments [49].

#### 4.3.2. Deep Fusion Architectures for Environmental Robustness

Fusion strategies are also tailored to specific traffic management applications. Combining mmWave radar with cameras improves traffic incident detection, enabling cross-validation of events and mitigating the impact of visual obstructions such as fog or low visibility [37]. In smart parking scenarios, hybrid fusion approaches allow low-power radar sensors to activate high-power cameras only upon motion detection, reducing energy consumption while maintaining reliable occupancy monitoring [50].

### 4.4. Alternative Sensing Modalities

While vision and radar dominate high-speed arterial monitoring, they often present prohibitive power and bandwidth requirements for widespread deployment in remote or resource-constrained environments. Consequently, infrastructure designers are increasingly adopting alternative sensing modalities tailored for specific operational needs from low-power environmental monitoring to acoustic density detection to ensure comprehensive network coverage without excessive capital expenditure.

#### 4.4.1. LoRaWAN for Environmental Monitoring

Beyond traffic flow, RSUs are increasingly tasked with environmental monitoring to support “Green Routing.” LoRaWAN IoT systems integrated into traffic monitoring stations enable the cost-effective measurement of air parameters (PM2.5, CO, NO_2_) over long ranges with minimal power consumption. By correlating air quality data with traffic density, RSUs can dynamically reroute heavy goods vehicles away from pollution hotspots [42]. However, from a security perspective, while LoRaWAN offers efficiency, its low bandwidth makes it susceptible to “Replay” and “Bit-Flipping” attacks, where falsified air quality reports could maliciously trigger city-wide traffic diversions.

#### 4.4.2. Fiber Optic and Infrared Traffic Sensing

Certain traffic monitoring environments impose constraints that necessitate alternative sensing configurations. Combining wireless radar sensor networks with Bragg grating optical fiber sensors allows for the measurement of vehicle weight and wheelbase at border crossings, enabling infrastructure-based classification under controlled entry conditions [51]. In tunnel environments where radar multipath effects degrade measurement accuracy, dual-infrared laser systems offer a reliable alternative for speed enforcement [52].

For developing economies, cost-effective alternatives to camera networks are required. Systems utilizing Infrared (IR) sensors combined with Load Cells offer a robust solution for traffic density detection. IR sensors detect vehicle presence, while load cells classify vehicle types based on weight, enabling dynamic signal timing optimization without the need for high-bandwidth video transmission [53].

Not all smart intersections require expensive vision systems. Density-based traffic signal systems utilizing ultrasonic sensors provide a cost-effective alternative for developing nations. By measuring the physical density of vehicles in a lane via sound wave reflection, these systems dynamically adjust green times without the privacy concerns or high bandwidth costs of video analytics [41]. From a security standpoint, while ultrasonic sensors are immune to visual adversarial patches, they are vulnerable to Audio Jamming attacks that can artificially trigger max-green phases.

## 5. Intelligent Traffic Control and Management

The aggregation of high-fidelity sensor data is futile without intelligent mechanisms to interpret and act upon it. Traffic signal control is currently undergoing a paradigm shift, moving away from rigid, pre-programmed timing plans toward adaptive, data-driven logic. This section explores the evolution of control hierarchies, tracing the trajectory from legacy hardware constraints to the deployment of Digital Twins and Generative AI, which promise to manage the unpredictable nature of mixed-autonomy traffic flows.

**Figure 5:**
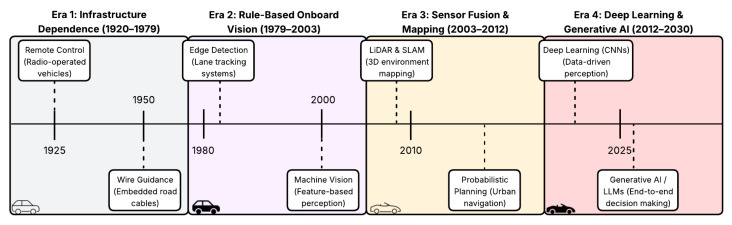
Technological evolution of autonomous vehicles categorized by dominant control paradigms, from infrastructure-dependent automation to learning-based and generative intelligence.

Figure 5 illustrates four major paradigms in the development of autonomous vehicle control. Early systems were infrastructure-dependent, embedding “intelligence” in the roadway through external guidance mechanisms. This progressed to rule-based onboard vision, where vehicles relied on deterministic algorithms to track visual features such as lane markings. The subsequent era introduced probabilistic robotics, enabling navigation in unstructured environments through sensor fusion and statistical mapping. The current era, catalyzed by the success of deep learning in 2012, represents a fundamental shift toward data-driven and generative control, where end-to-end models learn control policies directly from data rather than explicit programming.

### 5.1. Hardware Evolution: From PLCs to Embedded Edge AI

While AI provides the logic, the physical execution relies on robust hardware. Hybrid architectures combining Programmable Logic Controllers (PLCs) with Microcontrollers offer a centralized control solution that balances industrial reliability with computational flexibility for monitoring systems [54]. On the software side, Artificial Neural Networks (ANNs) are now capable of generating real-time signal timing designs by directly utilizing surveillance data from Smart Intersections, bridging the gap between perception and actuation [55]. However, the introduction of complex microcontrollers into legacy PLC cabinets expands the cyber-attack surface, necessitating firmware-level verification to prevent “Logic Bomb” injections that could freeze intersections in hazardous states. To optimize public transport reliability, machine learning models (Logistic Regression, Gradient Boosting, SVM) are applied to estimate real-time traffic density using attributes like speed, longitude, and geohash. These models improve bus arrival time predictions, directly enhancing the efficiency of the public transit network [56].

### 5.2. Simulation and Digital Twins

While early Digital Twins focused on traffic flow, recent systematic reviews highlight their critical role in Operation and Maintenance (O&M). Digital Twin (DT) frameworks now integrate structural health monitoring data to predict infrastructure failures (e.g., bridge fatigue, signal degradation) before they occur, shifting maintenance from reactive to predictive [57]. Running these city-scale Digital Twins requires immense computational power. Parallel algorithms implemented on supercomputers are necessary to process microscopic traffic flow models in real time. By decomposing the traffic network into sub-domains processed by parallel Graphics Processing Unit (GPU) clusters, simulation speeds can exceed real-time requirements, allowing the DT to “fast-forward” and predict congestion hours in advance [58].

#### 5.2.1. Digital Twins for Operations and Maintenance (O&M)

Digital Twins are evolving from passive monitors to active Co-Pilots for urban traffic. Conceptual models now utilize DTs to coordinate speed and signal phases in congested urban environments. By simulating the interaction between vehicle speed trajectories and signal timing in a virtual environment, the DT Co-Pilot provides real-time speed advisory commands to vehicles, harmonizing flow and reducing stop-and-go waves before they occur [59].

The scope of Digital Twins extends beyond city limits. In interurban scenarios, DT frameworks are deployed to manage Variable Speed Limits (VSL) and lane-changing guidance on highways, optimizing throughput during transition zones [60]. Furthermore, AI-enabled prediction systems like ASTRA demonstrate how Hotspot Prediction and resolution strategies used in En-Route Airspace can be adapted for managing complex traffic complexity, highlighting the convergence of ground and aerial traffic management logic [61]. The reliability of alert systems ultimately depends on the physical condition of roadside assets themselves. AI-based video analysis automates the detection of damaged signage and roadway surface defects, reducing reliance on manual inspection and enabling continuous infrastructure monitoring [62]. Complementing visual inspection approaches, Weibull distribution modeling predicts the structural failure of highway sign supports, allowing transportation agencies to prioritize maintenance and replacement decisions proactively based on quantified risk [63].

#### 5.2.2. Parallel Intelligence and Parallel Simulation Frameworks

Scalability remains the primary bottleneck for city-wide Digital Twins. Parallel multi-level simulation frameworks address this by coupling macroscopic models (for network-wide flow) with detailed microscopic models (for individual intersection dynamics). This hybrid approach allows for the large-scale testing of intelligent transportation systems without the computationally prohibitive cost of simulating every vehicle microscopically at all times [64].

Fundamentally, this approach relies on the ACP methodology (Artificial systems, Computational experiments, and Parallel execution). Wang et al. introduce the concept of decentralized autonomous organization-based traffic control, in which parallel intelligence leverages foundation models to manage transportation infrastructure as interacting social agents [65]. Building upon this theoretical framework, Tian et al. implement an active control methodology grounded in parallel control theory [13]. Their approach constructs an artificial traffic system that operates in parallel with the physical environment, enabling the simulation and evaluation of alternative signal strategies using predicted traffic states. Optimized control policies generated within the artificial layer are then proactively deployed in the real system, resulting in substantial reductions in travel delay compared to conventional reactive control schemes [13].

**Figure 6:**
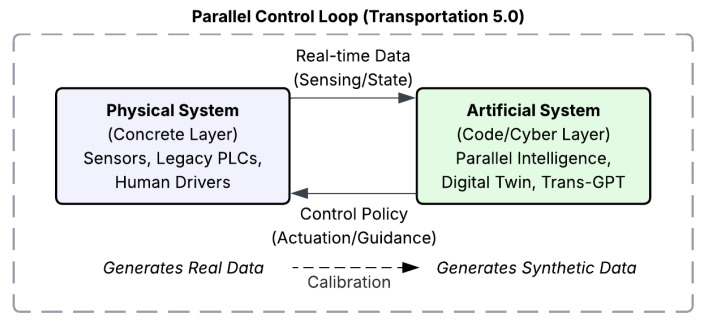
Parallel intelligence loop linking physical infrastructure with a continuously updated digital twin for policy optimization. Solid arrows indicate real-time data and control exchange, while the dotted arrow denotes data calibration. The outer dashed border represents the unified Transportation 5.0 framework.

Addressing the security implications of such tightly coupled cyber-physical environments, Lv et al. employ digital twins integrated with CNN–SVR models to predict and assess security threats in real time, thereby enhancing the resilience of cooperative intelligent transportation systems [66]. Figure 6 conceptualizes the parallel intelligence framework in which a digital twin operates continuously alongside the physical transportation system. Real-time sensing data are mirrored into an artificial system, where generative modeling expands sparse observations and supports rapid policy testing. The resulting control guidance is then deployed back to the physical layer, forming a closed-loop process of calibration and optimization rather than a one-time offline simulation.

While the embedded hardware platforms and programmable logic controllers discussed in Section 5.1 provide the industrial reliability required for physical actuation, their operation remains fundamentally constrained by rigid, rule-based logic and preconfigured timing plans [54]. Such deterministic architectures lack the computational flexibility necessary to manage the dynamic and highly contextual nature of mixed-autonomy traffic, where interaction patterns, behavioral uncertainty, and edge cases routinely exceed the expressive capacity of static control rules. Although the digital twin frameworks reviewed in Section 5.2 enhance situational awareness through predictive simulations and fast-forward analysis, they primarily function as passive evaluators rather than autonomous decision-makers. Digital twins can forecast congestion and system degradation, but they do not inherently reason about novel scenarios, semantic context, or intent-driven trade-offs in real time.

This gap between reliable physical execution and predictive simulation motivates a shift toward generative control paradigms capable of higher-level reasoning. Control frameworks are therefore transitioning beyond numerical optimization toward models that can interpret complex traffic scenarios, synthesize contextual information, and generate adaptive strategies under uncertainty. This evolution, often conceptualized as Transportation 5.0, leverages generative intelligence and Large Language Models to enable infrastructure to reason over varied inputs using semantic representations and natural language abstractions, thereby bridging the divide between static hardware control and dynamic, cognition-driven traffic management [65,67].

### 5.3. Generative AI and Large Language Models (LLMs) in Control

As introduced in the preceding sections, the transition to Transportation 5.0 and Parallel Intelligence necessitates a shift from rule-based logic to generative control paradigms [65]. To avoid conceptual redundancy, this section focuses strictly on the operational execution of these frameworks via LLMs. Rather than acting merely as digital twins, LLMs are increasingly utilized to generate active traffic signal control strategies through natural language interaction, fundamentally blurring the boundary between human intent and automated infrastructure control [67].

#### 5.3.1. Natural Language as Policy: LLMs for Signal Coordination

Beyond simulation-based optimization, generative artificial intelligence is increasingly investigated as a direct mechanism for traffic control logic generation. Recent studies demonstrate that Large Language Models can be used to derive arterial signal coordination strategies through natural language interaction, showing that language-based policy representations are capable of producing effective green wave coordination across urban corridors. In parallel, generative adversarial learning has been integrated into parallel training frameworks to augment data for deep behavioral cloning, allowing traffic control agents to learn and reproduce decision patterns traditionally crafted by human traffic engineers under complex and dynamic traffic conditions [68].

Despite their expressive capability, the deployment of foundation models on roadside infrastructure remains constrained by fundamental edge computing limitations, particularly memory bandwidth and autoregressive inference latency. Large-scale language models with billions of parameters frequently exceed the static RAM (SRAM) and memory bandwidth capacities of standard Roadside Units.

The dynamic accumulation of Key–Value (KV) caches during extended context processing makes real-time token generation fundamentally incompatible with the millisecond-level actuation requirements of safety-critical signal control [11]. Furthermore, the operational feasibility of deploying foundational models at the edge is severely constrained by thermal limits and power consumption. Unlike legacy PLCs (Transportation 1.0) that operate reliably under extreme environmental conditions with minimal power, the continuous inference of Generative AI requires specialized cooling and energy infrastructure, potentially undermining the sustainability goals of modern infrastructure. To address this mismatch between strategic intelligence and real-time responsiveness, recent system designs adopt a hierarchical decoupling of control responsibilities. Within this architecture, a high-level meta-policy (the Strategic Layer), typically implemented using a global language model, operates at a coarse temporal scale to generate long-horizon semantic objectives. These objectives are then delegated to lightweight, decentralized sub-policy agents (the Tactical Layer) that execute fine-grained phase adjustments at the intersection level. Evaluating these hierarchical approaches, Zhu et al. [69] demonstrate that global adversarial guidance can stabilize the meta-policy, whereas Shen [70] proves that localized reinforcement learning at the tactical layer is sufficient to ensure responsiveness to immediate traffic fluctuations, collectively preserving strategic coherence.

#### 5.3.2. Generative Models for Risk Recognition and Scenario Planning

Beyond traffic signal coordination, generative models are increasingly explored for risk recognition and scenario-level reasoning in intelligent transportation systems. Large Language Models can analyze a variety of inputs, including textual descriptions and vehicle telemetry, to identify hazardous driving contexts that may not be captured by rule-based logic. By interpreting unstructured scenario information, the model functions as a high-level reasoning component capable of anticipating potential collisions and unsafe interactions in complex traffic environments [71].

In addition, the probabilistic nature of generative models introduces the possibility of safety hallucinations, where a hazardous scenario may be misclassified as benign with high confidence. This limitation necessitates the integration of a deterministic Safety Assurance Layer to validate and constrain model outputs before execution. To address such failure modes, recent frameworks embed formal safety mechanisms, including Control Barrier Functions (CBFs) and neural Lyapunov certificates, directly into the policy architecture [72]. These safety assurance mechanisms act as a strict mathematical filter between the tactical layer and physical actuators. Specifically, a CBF defines a forward-invariant safe set H; if the generative tactical policy proposes an action that would drive the system state outside H (e.g., initiating a conflicting green phase), the CBF mathematically projects the action back into the safe operational space. Similarly, neural Lyapunov certificates guarantee asymptotic stability for learned controllers under dynamic traffic perturbations. Together, these techniques restrict the action space, ensuring that even if a generative model hallucinates an unsafe control decision, the physical execution layer enforces hard constraints [12].

As a complement to formal verification, physics-informed learning has emerged as a complementary strategy for improving robustness in generative control systems. By incorporating loss functions derived from traffic flow conservation laws, such as the Lighthill–Whitham–Richards model, learning-based policies can be regularized against data-driven anomalies and physically implausible behaviors [12]. Together, these approaches position generative models as strategic reasoning tools that operate within rigorously enforced safety and physical consistency boundaries.

**Figure 7:**
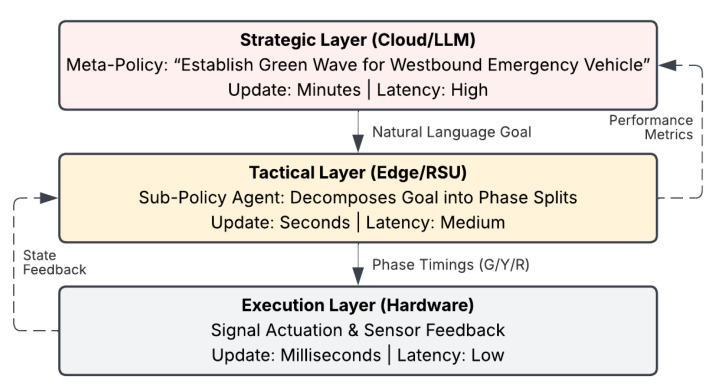
Hierarchical control architecture decoupling high-latency LLM-based planning from real-time execution at the edge and hardware layers. Solid arrows indicate the top-down flow of control directives, while dashed arrows denote the bottom-up flow of state feedback and performance metrics.

Figure 7 summarizes a practical strategy for applying foundation models to time-critical traffic control. High-latency LLM inference is confined to a strategic layer that outputs goal-level constraints, while an edge-level agent translates these directives into second- and millisecond-scale actuation commands. This decoupling preserves real-time safety requirements while enabling semantic, context-aware planning.

### 5.4. Network-Wide Coordination and Optimization

To link these isolated intersections, coordination models are essential. Mathematical models describing the “Start-Wave” dynamics of traffic flows allow for the precise calculation of offsets between coordinated traffic lights. By synchronizing the start-wave of downstream intersections with the arrival wave of upstream traffic, delays are minimized without complex adaptive logic, providing a robust baseline for city-wide coordination [73]. Validating these models requires high-fidelity data; online vehicle position information systems now provide the real-time Ground Truth necessary to validate simulation parameters dynamically, ensuring that the control logic does not drift from reality [74].

Achieving effective traffic signal control at the city scale requires coordination across intersections while addressing the computational challenges associated with high-dimensional decision spaces. To this end, hierarchical and game-theoretic formulations have been proposed to structure cooperation among distributed control agents.

Effective cooperation further depends on the integration of diverse data sources. Yang et al. propose a multi-agent reinforcement learning framework that incorporates artificial intelligence of things concepts to fuse data from distributed Internet of Things (IoT) sensors [75]. By constructing a unified state representation from diverse inputs, the approach enables coordinated decision-making based on network-wide situational awareness rather than isolated local observations [75].

Despite the growing dominance of data-driven and learning-based methods, mathematically grounded control models continue to play an important role in traffic signal optimization. Wang et al. apply adaptive linear quadratic regulator techniques to large-scale urban networks, capturing non-linear traffic dynamics while maintaining computational efficiency and interpretability [76]. Extending this work, bilinear system modeling is employed to represent complex interactions between traffic states and control actions, offering an analytically tractable alternative to black-box neural architectures for network-wide signal optimization [77].

#### 5.4.1. Game-Theoretic and Multi-Agent Reinforcement Learning

Shen et al. develop a hierarchical Nash–Stackelberg game model in which regional agents optimize macroscopic traffic states while intersection-level agents focus on local phase decisions. This leader–follower structure enables scalable multi-agent reinforcement learning by decomposing global optimization into coordinated subproblems [14]. In a related effort, Li et al. formulate network-level signal cooperation using higher-order conflict graphs, transforming the coordination problem into a maximum weight independent set formulation that captures deep structural dependencies across intersections [78].

#### 5.4.2. Cooperative Adaptive Cruise Control (CACC) for Platooning

Beyond intersection control, traffic optimization is moving toward vehicle-level coordination. CACC enables vehicles to form platoons, reducing aerodynamic drag and increasing road capacity. However, the presence of “unconnected” human-driven vehicles in mixed traffic streams introduces instability. Safety assessments utilizing high-fidelity simulations reveal that while CACC improves string stability, the unpredictable braking of unconnected vehicles can trigger shockwaves that disrupt the platoon, necessitating “Gap-Regulation” algorithms that dynamically increase spacing when non-connected vehicles are detected [79].

### 5.5. Multimodal Prioritization Strategies

Traffic incidents require rapid, coordinated responses to prevent secondary accidents. Automated approaches now generate and evaluate traffic incident response plans (IRPs) in real time. By simulating the incident impact, these systems automatically select the optimal combination of signal retiming, ramp metering, and detour messaging, removing the latency of human decision-making from the emergency loop [80].

A persistent challenge in urban signal control lies in balancing the immediate preemption needs of emergency vehicles and public transit with the progression requirements of general traffic flow. Traditional preemption strategies often prioritize emergency response at the expense of widespread congestion, motivating the development of more nuanced and adaptive prioritization mechanisms.

#### 5.5.1. Emergency Vehicle Preemption and Routing

Zhong and Chen propose a real-time signal control strategy based on on-demand signal timing, in which an emergency response level is computed dynamically to determine when and where priority should be granted. By selectively adjusting road saturation and forming green corridors only when required, the approach minimizes unnecessary disruption to non-emergency traffic while preserving rapid emergency response [81]. Adopting a learning-based perspective, Cao et al. introduce a deep reinforcement learning framework that simultaneously accelerates emergency vehicle passage and minimizes delay imposed on conflicting traffic streams [82]. Their gain-with-no-pain formulation outperforms greedy preemption approaches by explicitly accounting for the broader network impact of emergency prioritization [82].

#### 5.5.2. Public Transit Priority and Reliability Optimization

For public transit systems, the emphasis shifts from preemption toward active and continuous prioritization. Chen presents an adaptive multimodal signal control framework that provides concurrent progression bands for private vehicles and transit-oriented green phases for buses [83]. By dynamically adjusting cycle lengths and offsets in real time, the system ensures transit reliability without degrading overall corridor progression. At the design stage, Keblawi and Toledo introduce grammatical evolution techniques to automate the generation of actuated signal control logic. Rather than tuning predefined parameters, their approach evolves the structure of the signal plan itself, including phase composition and detector placement, enabling customized designs optimized specifically for transit priority [84].

The transition from conventional hardware to adaptive software is summarized in Table 4, which outlines the evolution of traffic control logic from Concrete to Code. The table traces the progression from Legacy (1.0) systems relying on fixed-timer PLCs to Adaptive (2.0) methods utilizing Deep Reinforcement Learning. Crucially, it characterizes the emerging Generative (3.0) era, where Large Language Models are employed as policy generators. This comparison emphasizes the shift in risk profile: while legacy systems faced physical wear, generative systems face probabilistic risks such as hallucinations regarding safety-critical decisions.

## 6. Data Integrity, Security, and Adversarial Resilience

As transportation infrastructure becomes increasingly software-defined and interconnected, the attack surface expands commensurately. Security in ITSs extends beyond traditional data confidentiality; it encompasses the critical assurance of data integrity and availability, where a millisecond delay or a single falsified sensor reading can result in physical harm. This section analyzes the cyber-physical threat landscape, focusing on how adversarial actors exploit the very intelligence designed to optimize the system.

### 6.1. The Dual Role of AI: Defender and Attack Surface

As AI-driven control and optimization become central components of ITSs, they simultaneously introduce new adversarial opportunities. While the security objectives of confidentiality, integrity, and availability remain fundamental, their realization within transportation systems is constrained by strict real-time and safety requirements.

### 6.2. Threat Landscapes in Intelligent Infrastructure

As transportation infrastructure becomes increasingly software-defined, the attack surface expands beyond traditional network intrusions to encompass the physical and cognitive layers of the system. Adversaries can now exploit the very intelligence designed to optimize traffic flow, utilizing vectors that range from physical sensor spoofing to subtle algorithmic manipulations. This section categorizes these emerging threats, distinguishing between attacks that blind perception systems and those that corrupt decision-making logic.

**Figure 8:**
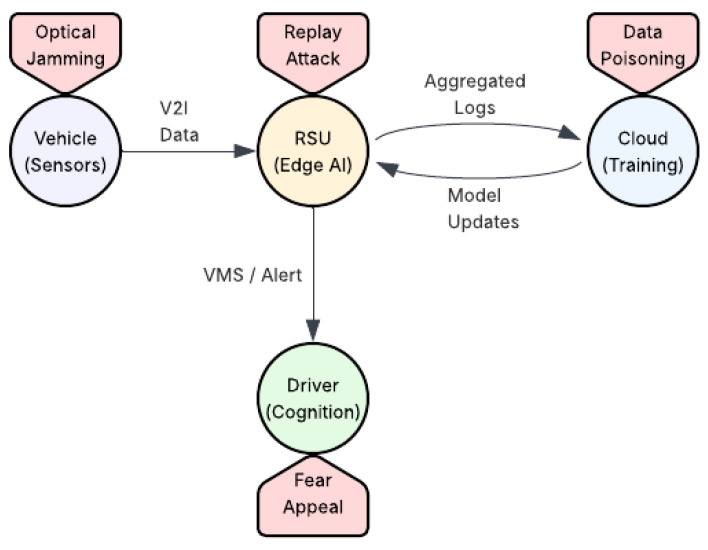
Analytical model of the cross-layer threat cascade. The diagram illustrates how adversarial influence propagates from physical sensing and vehicle data through edge intelligence and cloud learning to ultimately affect human cognitive responses.

Figure 8 illustrates a cross-layer attack surface in which adversarial influence is injected at multiple points along the cyber–physical–cognitive pipeline, spanning physical sensing, edge intelligence, cloud learning, and human cognition. The figure distinguishes attacks targeting sensor inputs, edge-level message integrity, cloud-side learning processes, and driver-facing information channels. Rather than isolated vulnerabilities, these attack points form an interconnected surface through which corrupted data and control decisions can propagate across the infrastructure loop. The diagram further emphasizes the propagation pathway highlighted in this section. Manipulation at the sensing or communication layers can distort the state observed by the RSU, influence model updates exchanged with the cloud, and ultimately affect the content of roadside messages and alerts delivered to drivers. In this way, low-level technical compromises can escalate into cognitive effects at the human interface, reinforcing the sensor-to-psychology scope of this survey and demonstrating how cross-layer failures translate into real-world safety risks.

#### 6.2.1. Data Poisoning and Backdoor Attacks on Learning Models

Modern Intelligent Transportation Systems increasingly rely on reinforcement learning and deep learning models for traffic signal control, transit prioritization, and autonomous decision-making. While these approaches provide adaptability in complex and dynamic environments, their dependence on data quality introduces fundamental vulnerabilities that can be exploited through the manipulation of input signals.

As ITSs rely on massive datasets for training, they become susceptible to Data Poisoning. A recent survey identifies that attackers can inject malicious perturbations into training datasets (e.g., traffic flow logs), leading to “Backdoor” vulnerabilities where the AI model performs normally under standard conditions but fails catastrophically when a specific “trigger” pattern is present [8]. This represents a “Sleep Agent” threat: the traffic control AI appears functional during testing but can be remotely sabotaged during peak hours via a specific sequence of vehicle inputs.

#### 6.2.2. Physical Layer Attacks: Optical Jamming and Spoofing

Adversarial attacks are moving from digital perturbations to physical execution. Simulations of attacks on Autonomous Vehicle perception systems demonstrate that simple physical tools, such as LED light strobes and color-light flashes, can blind traffic sign recognition models. By introducing Gaussian noise via strobing lights, attackers can force YOLO-based classifiers to misinterpret or ignore stop signs, proving that Optical Jamming is a viable, low-cost attack vector against Level 4 autonomous systems [15].

The effectiveness of reinforcement learning-based traffic control is tightly coupled to the accuracy and reliability of the observed state information. Manipulation of traffic flow measurements, including queue lengths and waiting times, can cause learned control policies to behave adversarially, resulting in intentional congestion rather than optimization. This risk is exemplified by adaptive controllers that rely on proximity sensor data to estimate traffic density; spoofed or falsified sensor readings in such systems directly corrupt the control logic and distort signal timing decisions [88]. Similar vulnerabilities arise in deep reinforcement learning models designed for transit prioritization, where falsified priority signals can trigger unwarranted preference for specific traffic streams, disrupting overall network efficiency without legitimate cause [89].

Table 5 maps the diverse threat landscape directly to the infrastructure layers defined in this survey. By correlating specific attack vectors, such as Replay Attacks at the network layer and Optical Jamming at the physical layer, with their consequences, the table demonstrates how cyber-threats translate into physical safety risks. It explicitly links abstract vulnerabilities, such as data poisoning in AI models, to tangible outcomes like autonomous vehicle blindness or signal manipulation, providing a structured view of the cross-layer attack surface.

#### 6.2.3. Adversarial Examples in Intrusion Detection Systems

As machine learning becomes integral to traffic system defense, security mechanisms themselves have emerged as high-value targets. Intrusion detection systems deployed within in-vehicle networks, such as the CAN bus, increasingly rely on learned models to identify malicious behavior, rendering them susceptible to adversarial manipulation [95].

Adversarial machine learning techniques can be used to generate carefully perturbed network frames that evade detection by state-of-the-art intrusion detection systems while retaining malicious functionality. By training substitute models on On-Board Diagnostics-II (OBD-II) data, black-box adversarial examples can be constructed that degrade the performance of advanced intrusion detection systems, reducing detection accuracy and effectively neutralizing internal vehicle security defenses. These findings highlight a critical weakness in AI-based defense strategies, where the defender’s learning capability can be exploited as an attack surface [96].

Data integrity within intelligent transportation systems is also influenced by underlying network conditions. Packet loss, jitter, and congestion can degrade the reliability of real-time data streams, leading to incomplete or misleading system states. Network-level impairments can affect perceived data quality, necessitating strategies such as LSTM-based active queue management to predict and mitigate queue delays [97]. While often overlooked in security contexts, preserving data integrity in traffic systems requires not only robust authentication mechanisms but also resilient network management to prevent information degradation during transmission.

### 6.3. Trust and Authentication Frameworks

Securing RSU networks requires a multi-layered approach that addresses device integrity, data confidentiality, and trust management without compromising latency.

A primary security challenge is preventing RSU impersonation. To address this, three-party authentication protocols based on Elliptic Curve Cryptography (ECC) have been developed. These models involve a trusted registration center to mediate between the vehicle and the RSU, preventing the spoofing attacks common in simpler two-party schemes [98]. In scenarios lacking infrastructure, anonymous identity-authentication schemes allow for secure 5G-V2V communication independent of fixed RSUs [99]. To reduce the latency overhead of Certificate Authorities, aggregation schemes are used where RSUs authenticate vehicles within localized “interaction zones” using lightweight ECDH key exchange [100].

Security must also extend to the data payload. Attribute-Based Encryption (ABE) allows for fine-grained access control, ensuring that only entities with specific attributes (e.g., Emergency Service or Original Equipment Manufacturer (OEM)) can decrypt specific vehicle data. Offloading the heavy computational burden of ABE to edge infrastructure secures the end-to-end link from the in-vehicle CAN bus to the cloud, balancing security with real-time performance requirements [101,102].

#### 6.3.1. Zero Trust Architectures and Attribute-Based Encryption

Modern frameworks are moving toward Zero Trust Architecture (ZTA) combined with real-time anomaly detection. Unlike perimeter-based security, ZTA assumes that no device, inside or outside the network, is trusted by default. By integrating machine learning (XGBoost/RF) with blockchain-based authentication, systems can detect anomalies in IoT data streams (e.g., traffic sensors) and automatically isolate compromised devices, mitigating threats like botnet injection in real time [90].

As ITS networks flood with Cooperative Awareness Messages (CAMs), batch processing becomes too slow for security. Streaming-based anomaly detection frameworks utilizing Enhanced Locally Selective Combination in Parallel Outlier Ensembles (ELSCP) allow for the on-the-fly verification of ITS messages. By analyzing the statistical properties of message streams in real time, these unsupervised models detect anomalies in vehicle position and speed reports immediately, preventing the propagation of false data across the V2X network [91].

#### 6.3.2. Blockchain and Decentralized Identity Management

To eliminate centralized points of failure, blockchain architectures are increasingly integrated into V2X. Multi-level blockchains, where RSUs act as cluster heads, provide a decentralized ledger for secure data transfer [103]. To resolve the scalability issues of linear blockchains, Bayesian Directed Acyclic Graph (DAG) models have been introduced. These allow Edge-RSUs to validate transactions in parallel, reducing the latency required for real-time vehicular networks [104].

#### 6.3.3. Physical Unclonable Functions (PUFs) for Hardware Security

Finally, the physical security of the RSU itself is paramount. Since RSUs are deployed in public spaces, they are vulnerable to capture. PUFs provide a hardware-based fingerprint that prevents cryptographic cloning even if the device is stolen [105]. Conversely, the vehicle fleet can act as a monitor for the infrastructure; frameworks using dashcams and YOLO models enable real-time anomaly detection of damaged roadside facilities [40]. To validate these physical and cyber defenses, Software-Defined Radio (SDR) frameworks are employed to audit V2X vulnerabilities at the signal level [106].

To counter manipulation, systems must rely on tamper-evident data sources. Data-driven analysis using Digital Tachograph (DTG) data provides a verifiable record of road traffic conditions. Unlike crowdsourced app data which is easily spoofed, DTG data is cryptographically signed at the hardware level within commercial vehicles, offering a high-integrity Ground Truth for traffic density and speed analysis [107].

Beyond individual vehicles and intersections, intelligent transportation systems increasingly depend on crowdsourced and cooperative data sources, including navigation platforms and V2X communication. While such data streams enhance situational awareness, they also introduce susceptibility to Sybil attacks, false event reporting, and coordinated misinformation.

To address trust and accountability in crowdsourced traffic systems, decentralized reputation management frameworks implemented through smart contracts offer a robust solution. These approaches assign trust scores to data contributors based on consistency with majority reports, executing reputation logic on a blockchain to ensure immutability and eliminate reliance on centralized authorities [108]. In vehicle-to-vehicle environments, the challenge of detecting insider threats posed by compromised but legitimate vehicles is addressed through collaborative intrusion detection frameworks based on distributed ensemble learning. Vehicles train local classifiers and exchange models with neighbors, evaluating their trustworthiness using statistical consistency checks to prevent poisoned models from influencing collective decision-making [109].

As learning-based traffic control systems become more pervasive, ensuring robustness to sensor failures, data manipulation, and privacy violations has emerged as a critical research focus. Jiang et al. address partial observability caused by sensor outages through a robust reinforcement learning formulation that employs low-variance state estimation, enabling reliable control even under blinded intersection conditions [110]. From a security perspective, Qiao et al. apply advantage actor–critic agents to identify and suppress spoofed sensor inputs during congestion attacks, thereby enhancing the resilience of phase optimization under adversarial conditions [111].

### 6.4. Privacy-Preserving Mechanisms

Efforts to secure data integrity often conflict with privacy preservation, particularly in systems that continuously collect fine-grained mobility information. Recent frameworks seek to reconcile these objectives by decentralizing learning while enforcing secure aggregation. Blockchain-enabled federated reinforcement learning architectures allow for secure model training in autonomous connected vehicle environments. In this architecture, vehicles train security and control models locally, preserving data privacy, while blockchain mechanisms are used to authenticate and aggregate model updates, mitigating risks associated with data poisoning and unauthorized manipulation. Extending these concepts to traffic signal control, federated proximal policy optimization frameworks enable intersections to collaboratively train control policies without sharing raw traffic flow data. This approach demonstrates not only enhanced privacy preservation but also improved convergence speed compared to isolated training approaches [112].

Preserving commuter privacy represents an equally important challenge. Ying et al. introduce a privacy-preserving traffic signal control framework that employs secure multi-party computation to train deep Q-network agents without exposing raw vehicle trajectory data [113]. Similarly, Liu et al. explore swarm learning approaches in 6G-enabled transportation systems, allowing decentralized agents to share model parameters rather than sensitive raw data, thereby balancing collaborative learning with privacy preservation [114].

#### Cluster-Based Federated Learning and Secure Aggregation

Standard Federated Learning (FL) can suffer from slow convergence in multi-vehicular networks. Adaptive Cluster-Based Federated Learning frameworks address this by grouping vehicles with similar characteristics (e.g., velocity, trajectory) into clusters. Anomaly detection models are trained locally within these clusters, and only model updates, not raw data, are shared. This approach improves detection accuracy for malicious messages while accommodating the non-IID (Independent and Identically Distributed) nature of vehicular data [92].

Crucially, clustering acts as a security containment strategy; if a specific cluster is poisoned by an attacker, the adaptive aggregation mechanism can isolate the corrupted model updates before they infect the global anomaly detection model.

## 7. Human–Infrastructure Interaction and Alert Systems

Regardless of the sophistication of upstream sensing and control algorithms, the human driver remains the final actuator in the majority of current traffic scenarios. Roadside alert systems and in-vehicle displays represent the primary interface between digital infrastructure and human agents. Consequently, the overall effectiveness of the cyber-physical system is bounded not only by the accuracy of the displayed information but also by how it is perceived, interpreted, and acted upon under real-world cognitive constraints [94,115]. This section traces the propagation of infrastructure decisions to the driver, examining the interface designs, reaction times, and psychological factors that govern effective human–machine interaction in connected environments.

### 7.1. Next-Generation Roadside Displays and Alerts

Roadside alert systems and displays represent the primary interface between digital transportation infrastructure and human drivers. The effectiveness of these systems depends not only on the accuracy of the information provided but also on how that information is perceived, interpreted, and acted upon under real-world driving conditions.

Beyond behavioral considerations, roadside display systems contribute measurable operational and economic benefits when deployed effectively. Evaluations of the financial impact of Variable Message Signs (VMS) quantify travel time savings during work zones and adverse weather conditions. Analysis demonstrates a consistently positive benefit–cost ratio, indicating that real-time route guidance provides substantial economic value to transportation networks [116]. However, the realization of these benefits depends critically on information accuracy. Frequent message phasing and prolonged update intervals degrade the accuracy of displayed travel times. Static messaging strategies during peak congestion periods can improve reliability and sustain driver trust [117].

As V2X architectures mature, both the volume and complexity of real-time travel information delivered to drivers continue to increase. If poorly designed, Human–Machine Interfaces (HMI) can introduce significant cognitive distraction. Research by Agrawal et al. highlights that evaluating the impact of real-time information must go beyond tangible travel-time benefits to explicitly factor in the cognitive burden imposed on the driver [118]. Utilizing objective psychophysiological metrics such as electroencephalograms (EEG), studies demonstrate that drivers exert significantly more cognitive effort to perceive and process information on complex routes compared to simpler freeway environments. To minimize this cognitive burden, findings from the Wyoming Connected Vehicle (CV) Pilot demonstrated that the readability of CV applications was highest when utilizing standardized, low-complexity visual formats, while actively avoiding continuous auditory warnings for non-critical advisories to prevent annoyance [119].

#### 7.1.1. Variable Message Signs (VMS) and Visual Salience

The visual design of VMS plays a central role in shaping driver comprehension and reaction time. Analyses of factors such as pictogram complexity and textual content demonstrate significant differences in comprehension between drivers and non-drivers. Findings indicate that standardized visual formats are essential for minimizing cognitive load and ensuring rapid interpretation across diverse user populations [20]. Dynamic visual features are commonly employed to attract driver attention, yet their effectiveness is highly sensitive to calibration. Investigations into the vehicle-by-vehicle impact of flashing message signs reveal that excessive flashing can increase distraction rather than compliance, underscoring the need for balanced visual stimulation to preserve sign effectiveness [19].

Furthermore, the design of these messages must strictly adhere to human factors limitations regarding message complexity. According to NHTSA guidelines for Driver-Vehicle Interfaces (DVI), visual messages must minimize the number of text lines to reduce glance durations, as excessive complexity directly increases the risk of secondary task distraction [120]. When immediate action is required, auditory messages are preferred over visual text; however, to prevent startle responses or annoyance that could compromise vehicle control, these auditory alerts must be carefully calibrated to an amplitude of 10 to 30 dB above the masked threshold of the cab environment. Additionally, as infrastructure-based warning systems expand, designers must actively manage message congruence between the Driver–Infrastructure Interface (DII) and the DVI to prevent contradictory instructions (e.g., a roadside sign advising one action while the vehicle dashboard commands another) which can severely delay perception–reaction times [119,121].

#### 7.1.2. AI-Driven Hazard Detection and Active Warning

Contemporary alert systems are increasingly shifting from passive information displays toward active, AI-enabled hazard detection and warning mechanisms.

On the infrastructure side, intelligent traffic light systems integrate computer vision to detect roadway incidents and dynamically adjust signal phases to prevent vehicles from entering affected lanes. This approach enables rapid, automated mitigation of localized hazards without direct human intervention [122]. In contrast, vehicle-centric architectures bypass fixed infrastructure altogether. AI-enabled Internet of Things frameworks utilize vehicle-mounted sensors to detect collisions and immediately transmit location data and visual evidence to emergency services through cloud platforms, enabling rapid response independent of roadside systems [123].

### 7.2. Vulnerable Road User Protection

Environmental hazard detection further extends the role of AI in roadside safety. Deep learning techniques identify slippery road surfaces through texture analysis, allowing warning systems to activate before loss-of-control events occur [124]. In a complementary vehicle-to-infrastructure interaction, real-time road sign recognition systems integrate computer vision with automated speed control, enabling direct enforcement of traffic regulations through physical speed limitation rather than advisory alerts alone [125].

#### 7.2.1. Systems for Scooters and Motorcycles

While much research focuses on cars, motorcyclists and scooters represent high-risk, low-protection road users that are frequently overlooked in traditional V2X safety standards and car-centric infrastructure design. To address this gap, AI-Based Driving Assistance Systems (AI-DAS) specifically designed for scooters utilize lightweight edge platforms (e.g., Raspberry Pi) to perform real-time lane line detection and pedestrian monitoring. These systems provide immediate audio-visual warnings to riders, extending the virtual protective envelope to two-wheeled vehicles [126]. Furthermore, analyses of accident data highlight the need for specific physical infrastructure adaptations tailored to these vulnerable users, such as high-friction pavement markings and specialized roadside barriers [127].

#### 7.2.2. Disaster Response and Evacuation Guidance

Beyond traffic accidents, ITS infrastructure is pivotal for Disaster Response. IoT-enabled frameworks integrate environmental sensors (seismic, flood) with traffic displays to manage mass evacuations. In the event of a natural disaster, these systems automatically repurpose Variable Message Signs and traffic signals to prioritize evacuation routes for emergency responders, providing a Green Corridor for life-saving operations [128].

### 7.3. Cognitive Factors and Driver Behavior

Regardless of the sophistication of sensing and control algorithms, the final actuator in the majority of current traffic scenarios remains the human driver. Consequently, the effectiveness of roadside infrastructure is bounded by human cognitive limitations and behavioral psychology. This subsection examines the interface between digital alerts and human cognition, investigating how message framing, trust calibration, and emotional response influence driver compliance and safety. Crucially, it must be acknowledged that “the driver” is not a monolithic entity. Cognitive load, perception–reaction times, and trust calibration vary significantly across different demographics. Factors such as driver age, driving experience, and cultural background heavily influence the perceived ease of use and susceptibility to fear-appeals, requiring alert systems to be dynamically adaptable rather than adopting a one-size-fits-all approach.

A foundational justification for connected infrastructure is its ability to warn drivers of hazards before they are visibly apparent. However, the time required for a driver to perceive and react varies drastically depending on the environment. As infrastructure interacts with conditionally automated vehicles (SAE Level 3), the time required for a driver to cognitively transition from a secondary task back to driving becomes a critical bottleneck. A comprehensive study by Demicoli et al. established that the traditional 2.5 s Perception–Reaction Time (PRT) used in standard road design is wholly inadequate for Level 3 environments, as the 85th percentile PRT rises to 4.23 s when drivers are distracted [129].

Conversely, when drivers are actively engaged in the driving task, V2X connectivity demonstrably improves reaction times. In a high-fidelity simulator study, Ali et al. found that drivers in connected environments respond significantly faster (1.13 s) to lane-changing requests compared to baseline conditions without V2X aids (1.59 s), drastically increasing cooperative behavior [130]. Furthermore, recent simulator testing evaluating human driving reactions to “invisible” V2X communications (e.g., warnings without a visibly apparent reason) corroborates that drivers generally respond safely, maintaining appropriate reaction times between 0.58 s and 0.69 s without erratic panic braking [131].

#### 7.3.1. Impact of Fear-Appeals and Information Trust

Beyond visual salience, the psychological content of safety messages influences driver behavior. Investigations into the impact of displaying cumulative fatality statistics on roadside signs utilize AI-based sentiment analysis of connected vehicle telemetry data. By analyzing indicators such as speed variation, hard braking, and lateral acceleration as proxies for emotional response, it is shown that fear-inducing messages often trigger anxiety-related behaviors rather than cautious driving, revealing a counterproductive fear-appeal effect [94]. Synthesizing these behavioral dimensions, trust is identified as the dominant factor governing driver compliance. When drivers perceive information as outdated or unreliable, adherence declines sharply regardless of visual clarity or message prominence [115].

To combat this erosion of trust and prevent fear-appeals, infrastructure and in-vehicle systems must employ “graded” human-centered alerts. As highlighted by Zhang et al., cooperative safety intelligence fundamentally transforms safety into a collective intelligence problem [132]. To maintain human trust, alerts should follow a staged escalation: inform (contextual cue), warn (focused highlight), and command (explicit instruction). By conveying Predictive Quality of Service (PQoS) metadata, such as the confidence level of the prediction, the system allows the driver to accurately calibrate their trust, minimizing psychological degradation during inevitable false positives.

The mitigation of these fear-appeals is central to establishing driver trust, which is the primary mediating factor in the adoption of connected infrastructure. Applying the Technology Acceptance Model (TAM) to 5G Connected Autonomous Vehicles (CAVs) reveals that driver trust is heavily reliant on “Perceived Usefulness” (the belief that the system enhances driving performance) and “Perceived Ease of Use” (the belief that interacting with the system is effortless) [133]. Furthermore, “Perceived Compatibility”, how well the automated alerts fit into the driver’s existing values and experiences, has a direct positive influence on the behavioral intention to comply with infrastructure commands [134]. Therefore, if a roadside alert system is overly complex, it imposes a high cognitive burden that directly violates the system’s perceived ease of use [118]. Furthermore, frequent false positives or continuous, non-critical auditory warnings actively induce driver annoyance and violate perceived usefulness, rapidly eroding trust and causing drivers to systematically ignore future cooperative warnings [119,132].

#### 7.3.2. The Gap Between Automated Enforcement and Human Compliance

Some approaches propose removing the human from the control loop altogether through automatic enforcement mechanisms, such as direct speed limitation based on sign recognition [125]. However, such interventions raise unresolved questions regarding accountability and liability. If an automated traffic light or incident detection system intervenes to prevent a collision and fails due to sensor or model error, responsibility becomes ambiguous. Resolving this handoff between human judgment and machine autonomy represents a critical challenge for future transportation governance.

Overall, these findings show that the effectiveness of intelligent transportation infrastructure is not solely determined by sensing accuracy or algorithmic performance. Instead, system success ultimately depends on how information is perceived, trusted, and acted upon by human drivers operating under real-world cognitive constraints.

## 8. Synthesis: Cross-Layer Vulnerabilities and Interdependencies

The preceding sections have analyzed infrastructure components, sensing, networking, control, and human factors, largely in isolation. However, in a deployed Cyber–Physical System, these layers are tightly coupled, creating complex interdependencies where failures can cascade vertically through the stack. This section synthesizes the reviewed literature to map these cross-layer vulnerabilities, demonstrating how physical layer attacks propagate to logical control failures and ultimately manifest as hazardous human behaviors.

### 8.1. The Feedback Loop: Sensing Errors Leading to Actuation Failures

The convergence of Roadside Units, radar sensing, traffic control systems, data integrity mechanisms, and alert infrastructures reveals a tightly coupled ecosystem in which advances at one layer frequently introduce new vulnerabilities at another. Rather than operating as independent subsystems, these components form a feedback-driven architecture in which sensing, decision-making, communication, and human response are deeply interdependent.

A critical cross-layer vulnerability emerges at the intersection of perception and automated actuation. Reinforcement learning agents are widely deployed to optimize traffic signal timing and mitigate congestion by adapting to real-time conditions [135]. However, defense mechanisms designed for these controllers, such as the BlindLight framework proposed by Jiang et al. [110], must evolve beyond simple resilience to random sensor noise to explicitly counter intentional data poisoning strategies described in recent reinforcement learning analyses [9].

When physical sensing inputs, such as radar-based vehicle localization [47], are jammed or spoofed, erroneous state information propagates upward through the RSU and corrupts these edge-level control policies. Crucially, this corrupted logic is subsequently transformed into driver-facing alerts through roadside displays. As demonstrated by Okaidjah et al., when misleading or alarming messages reach Variable Message Signs, they trigger maladaptive driver responses, inducing abrupt braking and anxiety-driven behaviors rather than safe compliance [94]. This end-to-end interaction reveals a dangerous feedback loop where physical layer attacks bypass digital encryption to manifest as psychological impacts on human drivers, effectively turning the infrastructure’s intelligence against its users.

### 8.2. Analytical Formalization of the End-to-End Threat Cascade

To rigorously structure the conceptual “End-to-End” framework, we formalize the cross-layer threat model by defining the infrastructure as a sequence of interdependent transformation functions. Let the state of the infrastructure at time *t* be represented across three primary domains: the physical sensing layer S(t), the edge AI and control layer E(t), and the human cognitive layer C(t) [65,132].

Under benign operational conditions, the system functions as a deterministic mapping:

(1) $$E\left(t\right)=f_{edge}\left(S\left(t\right)\right),\;C\left(t\right)=f_{cog}\left(E\left(t\right)\right)$$

where $f_{edge}$ represents the AI-driven control logic (e.g., traffic signal phase timing) and $f_{cog}$ represents the driver’s behavioral response to the resulting infrastructure cues (e.g., Variable Message Signs and automated alerts) [65].

To substantiate the infrastructure threat cascade, we formally define the adversarial taxonomy and dynamics across three distinct stages. First, Propagation (*P*) is defined as the successful transfer of an adversarial perturbation δ across a layer boundary without sanitization. If an attack, such as optical jamming [15], is injected at the sensing layer such that the perturbed state is $S^{\prime }\left(t\right)=S\left(t\right)+\delta$, propagation occurs if the edge verification function $V_{edge}$ fails to reject it. Formally:

(2) $$P=1⟺V_{edge}\left(S^{\prime }\left(t\right)\right)=\mathrm{True}$$

Second, the Cascade (*K*) represents the transformation and potential amplification of the propagated error as it passes through the system’s decision logic [8]. The cascade is formally defined as the mathematical deviation in the edge state output:

(3) $$K=f_{edge}\left(S\left(t\right)+\delta \right)-f_{edge}\left(S\left(t\right)\right)$$

A cascade is deemed successful if *K* exceeds the system’s safe operational threshold, thereby corrupting the actuation layer.

Finally, the Cognitive Consequence (ΔC) captures the ultimate, measurable degradation in human performance or safety resulting from the cascaded error. This is quantified by changes in behavioral metrics, such as increased Perception–Reaction Time (PRT) [129] or the induction of anxiety-driven braking [94]:

(4) $$\Delta C=f_{cog}\left(E\left(t\right)+K\right)-f_{cog}\left(E\left(t\right)\right)$$

To illustrate this cascade with a concrete scenario, consider an optical jamming attack on a roadside camera. The initial physical perturbation δ manifests as targeted LED strobing that blinds the sensor [15]. The propagation P=1 occurs if the edge verification function fails to reject the corrupted frame. The cascade *K* is the resulting error in the edge logic, for example, failing to detect a pedestrian and erroneously commanding a green light phase. The cognitive consequence ΔC is the subsequent degradation in human safety, such as an unexpected hazard that forces the driver’s perception–reaction time to jump from a connected baseline of 1.13 s to an unsafe 4.23 s [129,132].

This analytical model proves that an infrastructure threat cascade is not merely a localized hardware failure, but a composite function where a physical-layer perturbation fundamentally alters the cognitive mapping.

### 8.3. The Latency–Security Trade-Off in Real-Time Systems

A persistent tension is also evident between latency constraints and cryptographic security requirements. Advanced traffic control strategies, including swarm learning-based coordination, depend on millisecond-level updates to maintain synchronized signal progression and responsive control. In contrast, robust RSU security architectures rely on computationally intensive cryptographic techniques such as attribute-based encryption to enforce fine-grained access control. To reconcile these competing demands, emerging approaches increasingly favor hardware-assisted authentication. PUFs enable secure device identification without incurring the computational overhead associated with complex cryptographic operations, thereby narrowing the gap between real-time safety requirements and strong security guarantees.

### 8.4. The Exploration vs. Safety Dilemma in Reinforcement Learning

A recurring tension exists in the deployment of Reinforcement Learning (Section 5). To find optimal policies, RL agents must “explore” the action space, occasionally taking suboptimal actions to learn. Khaleel and Ballagi highlight that in Autonomous Vehicle contexts, standard exploration techniques (like Epsilon-Greedy) are dangerous because a “random” exploration step could lead to a collision [86]. This creates a critical security gap: if an attacker can manipulate the Exploration Rate hyperparameter of a live traffic controller, they can force the system into a Safe-Fail mode where it randomly cycles signals under the guise of learning, causing chaos.

## 9. Open Challenges and Future Directions

The transition from theoretical frameworks to deployed intelligent infrastructure is constrained by technical, physical, and regulatory boundaries that current research has yet to fully resolve. While the AI-driven methodologies analyzed in the preceding sections offer substantial performance improvements, their integration into safety-critical environments introduces systemic risks that existing governance models cannot yet accommodate. Furthermore, the scope of transportation infrastructure is physically expanding beyond the roadway into the aerial domain, while the computational demands of next-generation digital twins are rapidly approaching the limits of classical edge hardware. This section identifies the critical open challenges, spanning regulatory compliance, multidimensional traffic management, and post-silicon computing, that must be addressed to realize the vision of Transportation 5.0.

As summarized in Table 6, we have categorized these critical open challenges into five primary domains to provide a centralized roadmap for future research. This table explicitly maps the core limitations of current systems, spanning regulatory compliance, multidimensional traffic management, post-silicon computing, public trust, and simulation fidelity, directly to the required future work necessary to realize the vision of Transportation 5.0.

### 9.1. Regulatory Paradox: Generative AI vs. Deterministic Safety Standards

The integration of Generative AI and LLMs into intelligent transportation infrastructure introduces a fundamental conflict with the regulatory foundations upon which modern safety standards are built. Existing certification frameworks were developed for deterministic, traceable control systems, where failure modes can be enumerated, tested, and bounded. In contrast, generative models exhibit probabilistic behavior, non-repeatable outputs, and opaque internal representations, creating a regulatory mismatch that current standards are not designed to resolve [65].

Functional safety standards such as ISO 26262 mandate rigorous risk assessment procedures based on quantifiable failure rates and Automotive Safety Integrity Levels (ASIL) [2]. These processes assume that system behavior can be exhaustively characterized and validated against deterministic specifications. However, LLM-driven control modules cannot be evaluated using conventional fault tree analysis, as identical inputs may yield divergent outputs across inference instances. As noted by Liu et al., existing certification pipelines are structurally incapable of validating black-box neural architectures against ASIL-D requirements without new mathematical frameworks for stability analysis [72].

Similarly, the Safety of the Intended Functionality (SOTIF, ISO 21448) framework was introduced to address performance limitations of perception systems [3]. While SOTIF expands the regulatory lens beyond component failure, it remains grounded in sensor insufficiency and foreseeable misuse [5]. It does not yet account for “hallucination” phenomena inherent to generative models, where internally plausible but externally incorrect representations are synthesized without sensor faults. For instance, LLM-based risk recognition systems may confidently misclassify a hazardous scenario due to training data bias rather than sensor noise, introducing a failure class that defies current safety envelopes [71].

At the communication layer, IEEE 1609.2 standards provide strong cryptographic guarantees for message authenticity [4]. These mechanisms secure the transmission channel but remain agnostic to the semantic correctness of transmitted content. A message generated by a compromised generative model may remain fully compliant with cryptographic validation while conveying contextually coherent yet hazardous guidance. As highlighted in recent data poisoning surveys, no current regulatory framework defines semantic security constraints capable of bounding such cognitively deceptive outputs [8].

Collectively, these limitations expose a regulatory paradox. Resolving this conflict requires regulatory evolution beyond static code inspection. Future compliance regimes must formalize the concept of safe envelopes; deterministic supervisory layers that enforce invariant system properties and ensure that learning-based components remain subordinate to certifiable control logic.

### 9.2. Urban Air Mobility (UAM) and the Vertical Expansion of Infrastructure

The definition of “Roadside” is expanding vertically to include low-altitude airspace. As Urban Air Mobility (UAM) matures, the management of aerial traffic will rely on protocols adapted from aviation. Analyses of Controller-Pilot Data Link Communications (CPDLC) reveal that digital text-based instructions are replacing voice comms for efficiency. However, the current unencrypted nature of legacy CPDLC links poses a severe risk; in a UAM environment, unauthenticated instructions could be injected to hijack drone trajectories, necessitating the migration to “Secured-CPDLC” standards for urban deployment [136]. Crucially, this aerial expansion must remain integrated with the ground-level End-to-End cascade. An attack on a flying RSU’s CPDLC link does not remain an isolated aerial event; corrupted drone trajectories or grounded drone swarms can directly cascade into ground-vehicle routing failures and pedestrian safety hazards, unifying the 3D airspace and terrestrial roadways into a single, interdependent threat surface.

#### 9.2.1. UAVs as Flying Roadside Units

Unmanned Aerial Vehicles (UAVs) are transitioning from hobbyist devices to critical ITS infrastructure. “Forerunner UAVs” can fly ahead of emergency responders to provide real-time video feeds and accident analysis, effectively extending the eyes of the control center beyond physical obstructions. This aerial perspective is crucial for managing complex incidents where ground sensors are occluded [140]. Furthermore, predictive modeling used in aviation, such as machine learning classifiers for “Go-Around” occurrences, can be adapted for UAM traffic management, predicting collision risks in 3D airspace before they materialize [141].

#### 9.2.2. Flight-Centric Control and 3D Security Challenges

In the domain of Urban Air Mobility (UAM), the traditional “Sector-Based” control model is becoming obsolete. The industry is shifting toward “Flight-Centric” Air Traffic Control (ATC), where controllers are assigned to specific aircraft rather than geographical sectors. This necessitates the development of “Allocation Centers” capable of dynamically balancing controller workload based on 4D trajectory complexity rather than spatial boundaries. This shift requires a complete redesign of the communication architecture to support seamless, sectorless handovers [137].

### 9.3. Next-Generation Computing Paradigms

The computational demands of city-scale digital twins, 6G signal processing, and real-time cryptographic verification are rapidly approaching the limits of conventional silicon-based architectures. To sustain the evolution of intelligent infrastructure, research is expanding into novel computing paradigms. This subsection reviews emerging hardware frontiers, including quantum and neuromorphic computing, which offer the potential to resolve the latency and optimization bottlenecks inherent in classical edge computing platforms.

#### 9.3.1. Quantum Machine Learning and Quantum-at-the-Edge

As data complexity exceeds classical computing limits, Quantum Machine Learning (QML) is emerging as a frontier technology. Quantum-enhanced Support Vector Machines (QSVM) have been applied to detect anomalies in Electric Vehicle (EV) battery systems with 98.7% accuracy. By mapping complex voltage/temperature correlations into high-dimensional quantum Hilbert spaces, these models can identify subtle degradation patterns that classical algorithms miss. Future research must explore “Quantum-at-the-Edge,” investigating if simplified quantum circuits can be deployed on RSUs to secure cryptographic keys against quantum-decryption attacks [138].

#### 9.3.2. Neuromorphic Processing for 6G Networks

Looking ahead, the transition toward sixth-generation communication networks and neuromorphic computing architectures introduces both opportunities and complexity. Spiking neural networks have been proposed for efficient radar signal processing [44], but their integration into RSU platforms raises fundamental challenges related to synchronizing asynchronous spike-based computation with time-slotted 5G and 6G communication protocols. In parallel, the increasing emphasis on non-terrestrial networks in emerging 3GPP releases positions satellite-assisted V2X connectivity as a critical component of future infrastructure [1]. Concepts such as satellite-based RSUs transition from theoretical constructs to practical necessities, demanding solutions for seamless handover, latency management, and coordination between orbital platforms and terrestrial traffic control systems.

### 9.4. Barriers to Adoption: Legal Liability and Public Trust

Human interaction with automated infrastructure remains an unresolved paradox. While roadside alert systems aim to guide driver behavior, empirical evidence shows that misinterpretation and mistrust persist.

Despite technical progress, non-technical barriers remain significant. The Decision-Making Trial and Evaluation Laboratory (DEMATEL) method reveals that “Legal Liability,” “Cybersecurity,” and “Public Trust” are the causal barriers hindering the widespread adoption of Autonomous Vehicles. Addressing these requires not just engineering solutions, but the development of explainable AI frameworks that can satisfy legal scrutiny and build public confidence [139].

### 9.5. Simulation and Validation Infrastructure for Transportation 5.0

A critical methodological gap identified in this survey is the continued reliance on single-domain simulation platforms to validate increasingly complex transportation systems. Traditional evaluation pipelines predominantly employ microscopic traffic simulators such as Simulation of Urban MObility (SUMO) and Verkehr In Städten - SIMulationsmodell (VISSIM), which are well-suited for modeling flow dynamics, intersection logic, and macroscopic congestion patterns. However, these platforms lack the sensory, physical, and cyber interaction fidelity required to evaluate adversarial perception failures, cross-layer interference, or generative decision instabilities.

As transportation infrastructure evolves toward Transportation 5.0 paradigms that integrate cyber–physical sensing, edge intelligence, and generative control policies, validation environments must reflect this multi-domain coupling. Emerging research increasingly adopts co-simulation frameworks that link traffic engines with physics-based rendering and robotics simulators. High-fidelity platforms such as Car Learning to Act (CARLA) and AirSim enable the photorealistic modeling of sensor modalities, environmental perturbations, and physical attack vectors, while traffic simulators coordinate large-scale vehicle interactions and infrastructure behavior.

This coupling is essential for experimentally evaluating adversarial scenarios. Because executing physical adversarial attacks (e.g., optical jamming or data poisoning) on live, real-world traffic intersections is prohibitively hazardous and ethically infeasible, validation must rely on high-fidelity virtual environments. For example, investigating optical attacks on roadside perception systems [15] requires environments capable of rendering physically grounded perturbations such as LED strobes, reflective textures, or adversarial signage while simultaneously maintaining realistic traffic flow. Single-domain simulators are fundamentally incapable of representing such cross-layer phenomena.

Future validation standards must therefore mandate co-simulation as a baseline methodology rather than an experimental enhancement. Without integrated cyber–physical testbeds, it is not possible to substantiate claims of robustness for generative transportation systems, nor to quantify resilience against sensor spoofing, model hallucination, and coordinated cyber–physical interference. Establishing standardized, multi-fidelity validation infrastructures will be a prerequisite for certifying Transportation 5.0 systems before real-world deployment. To overcome the prohibitive computational overhead of these hybrid co-simulations, future research must explore scalable architectures. Promising directions include cloud–edge split simulation, where computationally heavy physics rendering is offloaded to cloud clusters while traffic logic runs locally, and the development of AI-driven surrogate models capable of approximating high-fidelity sensor physics without the extreme rendering costs. Table 7 outlines the capabilities and limitations of current validation paradigms.

## 10. Conclusions

The transformation of transportation infrastructure from static Concrete assets into intelligent, Code-driven ecosystems represents a fundamental shift in how urban mobility systems are designed, operated, and secured. This survey has presented a comprehensive, End-to-End analysis of this evolution, tracing the flow of information from physical roadside sensing and communication layers, through edge-level intelligence and adaptive control, and ultimately to human interaction and cognitive impact. By synthesizing recent advances across hardware platforms, networking architectures, artificial intelligence models, and human-centered systems, this work highlights that Transportation 5.0 is not defined by isolated technological upgrades, but by the emergence of tightly coupled cyber–physical–cognitive infrastructures.

A central conclusion of this survey is the dual role of artificial intelligence within next-generation transportation systems. While deep reinforcement learning and generative models introduce advanced capabilities for managing chaotic traffic dynamics, cooperative control, and predictive optimization, they simultaneously expand the system attack surface. The migration from deterministic, rule-based controllers toward learning-driven and generative policies creates new vulnerability classes, including adversarial perception failures, data poisoning, and model hallucination, which cannot be addressed through traditional security mechanisms alone. These risks fundamentally reshape the threat landscape of smart infrastructure and demand security frameworks that explicitly account for probabilistic behavior and model uncertainty.

Equally critical is the growing disconnect between technological innovation and system validation. The survey reveals a persistent reliance on single-domain simulation environments that are unable to represent sensor-level attacks, physical perturbations, and cross-layer interference. As transportation infrastructure evolves toward deeply integrated cyber–physical systems, credible evaluation must move beyond traffic-flow correctness toward resilience under adversarial and environmental stress. Models that unify microscopic traffic modeling with high-fidelity physical and sensory environments emerge as a necessary foundation for future certification pipelines. Without such integrated validation infrastructures, claims of robustness for AI-driven transportation systems remain fundamentally incomplete.

This work further demonstrates that infrastructure security cannot be decoupled from human factors. Attacks targeting roadside sensing, communication, or display systems do not terminate at the digital boundary; they propagate through decision logic to influence human perception, trust, and behavior. Consequently, the security of Transportation 5.0 must be understood not only as a technical challenge, but as a cognitive one. Preserving system resilience requires architectures that maintain human trust, mitigate behavioral manipulation, and prevent the amplification of technical failures into physical or psychological harm.

Ultimately, realizing the vision of secure, AI-driven transportation demands unified system architectures that bridge hardware physics, algorithmic robustness, regulatory compliance, and human-centered design. Addressing these challenges will require coordinated advances in resilient edge intelligence, semantic security, cyber–physical validation platforms, and regulatory frameworks capable of governing non-deterministic control. Only through such integrative approaches can Transportation 5.0 mature into an infrastructure paradigm that is not only intelligent, but demonstrably safe, trustworthy, and sustainable.

## Figures and Tables

**Table 1:** Comparison of this work with recent related surveys (2022–2025).

Reference	Year	Focus Area	GenAI /LLMs	Physical Layer	Human Factors	Vertical Integration
Shaikh et al. [7]	2022	Swarm intelligence for traffic signal control	None (Evolutionary & swarm methods)	Abstracted (Assumes ideal sensor inputs)	Implicit (Traffic efficiency goals)	Algorithmic only (Control optimization)
Wang et al. [8]	2024	Data poisoning threats in ITSs	None (Adversarial ML focus)	Data-centric (Log injection, spoofing)	Risk-oriented (Impact discussion)	Data & model only (Security-specific)
Xiao et al. [9]	2025	Reinforcement learning for traffic control	Limited (Deep RL, not generative AI)	Abstracted (State-space representations)	Control-loop level only	Algorithmic only (Agent–environment loop)
Wu et al. [10]	2025	Digital twins for transportation infrastructure	None (Simulation-driven)	Modeled (Virtual replicas, BIM assets)	Operational (Maintenance, monitoring)	Digital–physical mirror (Asset modeling)
Zheng et al. [11]	2025	Edge large language models	Yes (Core focus)	Hardware-constrained (Latency, memory, power)	Application-level (Personal agents, services)	Partial (Compute & deployment stack only)
Di et al. [12]	2023	Physics-informed deep learning for traffic systems	Limited (GANs, hybrid learning)	Theoretical (Traffic flow laws, PDEs)	None	Partial (Theory & algorithm layer only)
This work	2026	Integrated AI-enabled transportation infrastructure	Core (LLMs & GenAI security)	Hardware-specific (Sensor physics, jamming, deployment)	Cognitive (Trust, fear appeals, behavioral response)	Full End-to-End (Physical → Edge AI → Human)

**Table 2:** Quantitative synthesis of validation environments and methodologies across the reviewed literature (n=140).

Domain	Count	Primary Approaches	Validation Environment	Ref. Studies
Traffic Control	n=45 (32.1%)	DRL, Parallel Control, LLMs	Micro-simulation (e.g., SUMO, VISSIM, CityFlow)	Tian et al. [13], Shen et al. [14]
Perception & Sensing	n=36 (25.7%)	YOLO, mmWave Radar, Fusion	Co-simulation/field data (e.g., CARLA), real-world feeds	Lin et al. [15], Kandavel et al. [16]
Edge/Network & Security	n=32 (22.9%)	SDN, DAG Offloading, ZTA	Network simulation (e.g., NS-3, OMNeT++), numerical (e.g., MATLAB)	Nkenyereye et al. [17], Guan et al. [18]
Human Factors & Alerts	n=27 (19.3%)	Alerts, VMS Design, Trust	Driving simulators/surveys (e.g., Unity3D), statistics	Basso et al. [19], Hernando et al. [20]

**Table 3:** Comparative analysis of roadside perception technologies: capabilities, limitations, and security risks.

Technology	Application	Key Advantage	Key Limitation	Representative Security Risk	Ref.
mmWave Radar	Traffic flow/speed	Robust under adverse weather and lighting conditions	Limited semantic resolution and object classification capability	Ghost targets via signal replay and RF spoofing	[35,37]
Photonic Radar	Precision tracking	High range resolution (cm-level) for fine-grained localization	Complex hardware integration and high deployment cost	Susceptible to wideband noise injection and radar jamming	[38]
Vision (YOLOv8)	Semantics/pedestrian detection	Rich semantic understanding (e.g., pedestrians, gestures, jaywalking)	Performance degradation in low-light and adverse weather	Adversarial patches, LED strobing, and optical spoofing attacks	[15,16,39,40]
Ultrasonic	Density and proximity detection	Low cost and scalable roadside deployment	Short detection range and environmental sensitivity	Acoustic and ultrasonic jamming via external emitters	[41]
LoRaWAN Sensors	Environmental monitoring	Low power consumption and long communication range	Low bandwidth and constrained payload size	Replay attacks and falsified data injection without strong authentication	[42]

**Table 4:** Evolution of traffic control logic: from deterministic to generative.

Paradigm	Core Tech	Decision Mechanism	Critical Limitation
Legacy (1.0)	PLCs/microcontrollers	Fixed-time plans; inductive-loop triggers	Rigid; vulnerable to logic manipulation and misconfiguration [54,85]
Adaptive (2.0)	Deep RL/optimization	Reward-driven phase selection and coordination	Exploration can create unsafe transient behaviors if not constrained [9,86,87]
Generative (3.0)	LLMs/GenAI copilots	Natural-language-as-policy; Hierarchical Meta-Policy/Sub-Policy	Hallucinations; Edge Inference Latency; Unverifiable reasoning without formal safety guards [11,59,65,67,69,72]

**Table 5:** Mapping cyber-physical threats to infrastructure layers.

Target Layer	Attack Vector	Mechanism	Physical/Cognitive Consequence
Physical sensing	Optical jamming/strobing	Injects structured noise (e.g., LED strobes) into camera pipelines	Mis-detection or blindness in traffic-sign/object recognition [15,48]
Network/RSU	Replay attack	Re-injects stale messages; exploits delayed freshness (AoI)	False historical vehicle states; degraded coordination and safety margins [26,90,91]
AI model	Data poisoning/backdoor	Inserts trigger patterns into training or updates	Model behaves normally until trigger appears, then misclassifies/redirects [8,92,93]
Human interface	Fear-appeal manipulation	Misleading warnings on VMS/alerts	Anxiety, panic braking, and unsafe driver responses [19,20,94]

**Table 6:** Summary of Open Research Challenges and Future Directions for Transportation 5.0.

Challenge Domain	Core Limitation/Research Question	Required Future Work	Section
Regulatory Paradox & Safety Standards	Existing functional safety standards (ISO 26262/SOTIF) are deterministic and cannot certify probabilistic generative AI [2,3].	Development of mathematically formal “safe envelopes” and supervisory layers to constrain learning-based components [72].	Section 9.1
Urban Air Mobility (UAM) & 3D Infrastructure	Unencrypted legacy CPDLC protocols are inadequate and vulnerable to hijacking in dense flying-RSU environments [136].	Transition to Secured-CPDLC standards and implementation of dynamic, flight-centric 4D trajectory allocation centers [137].	Section 9.2
Next-Generation Computing (6G & Edge)	Classical edge silicon cannot meet the immense processing and encryption demands of city-scale digital twins.	Integration of Quantum Machine Learning (QML) [138] and Neuromorphic (SNN) processing [44] to resolve bottlenecks.	Section 9.3
Liability, Governance & Public Trust	The handoff between machine autonomy and human judgment obscures legal liability when AI fails, hindering widespread adoption [139].	Creation of Explainable AI (XAI) frameworks that satisfy legal scrutiny and provide transparent accountability for autonomous actions.	Section 9.4
Simulation & Security Validation	Conventional simulators cannot model physical sensor spoofing, optical jamming, or adversarial perception failures [15].	Standardization of high-fidelity hybrid co-simulation platforms (e.g., CARLA + SUMO) for validating cyber-physical security.	Section 9.5

**Table 7:** Comparison of simulation methodologies for Transportation 5.0 validation.

Validation Paradigm	Primary Capabilities	Limitations for Transportation 5.0	Representative Platforms
Microscopic (traffic)	Traffic flow modeling, congestion dynamics, signal timing optimization, large-scale mobility patterns	Cannot represent sensor physics, perception uncertainty, spoofing behavior, or adversarial visual attacks	SUMO, VISSIM, CityFlow
Physics-based (robotics)	Photorealistic rendering, sensor modeling (LiDAR, camera, radar), vehicle dynamics, environmental interaction	Computationally intensive and difficult to scale to city-wide, multi-intersection traffic ecosystems	CARLA, AirSim, LGSVL
Co-simulation (hybrid)	**Emerging standard:** integration of traffic logic with high-fidelity physical and sensory modeling for cyber–physical evaluation	Complex time synchronization, co-scheduler design, and high computational overhead	CARLA + SUMO, VISSIM + Unity, AirSim + SUMO

## Data Availability

The data that support the findings of this study are openly available in Google Scholar.
